# Altruistic Giving Toward Refugees: Identifying Factors That Increase Citizens' Willingness to Help

**DOI:** 10.3389/fpsyg.2021.689184

**Published:** 2021-08-09

**Authors:** Dshamilja Marie Hellmann, Susann Fiedler, Andreas Glöckner

**Affiliations:** ^1^Social Cognition Center Cologne, University of Cologne, Cologne, Germany; ^2^Research Group Behavioral Law and Economics/Economic Cognition, Max Planck Institute for Research on Collective Goods, Bonn, Germany; ^3^Department of Strategy and Innovation, Vienna University of Economics and Business, Vienna, Austria

**Keywords:** refugee help, altruism, shared identity, prosociality, social closeness

## Abstract

Over the past decade, the world has faced an unprecedented refugee crisis. The large number of incoming refugees represents a challenge for host societies and its citizens triggering reactions from a supportive welcome to brusque rejection and hostile behavior toward refugees. In a pre-registered study, we investigated factors that could promote altruistic behavior in fully incentivized one-shot Dictator Game toward various receiver groups including refugees. We find that host citizens behave more altruistically toward refugees and other receiver groups if they (a) share a local identity with them (i.e., live in the same city), and (b) perceive them to be close (to the self) and warm-hearted. Moreover, citizens that are (c) generally more prosocial and hold a more left-wing political orientation are more willing to give. Unexpectedly, from a theoretical point of view, altruistic giving toward refugees was not influenced in the predicted direction by a shared student identity, competition and perceived income differences (although the latter effect was significant when considering all receiver groups). For shared student identity we even observe a reduction of altruistic behavior, while the opposite effect was predicted. We discuss implications for public policies for successful refugee helping and integration.

## Introduction

In the context of globalization and international migration, social interactions between people from different nations become increasingly important. Global challenges, such as climate change, war and conflict typically affect poorer countries most, causing large-scale migration. In Europe, Germany has been the country which received most refugees in recent years (United Nations Refugee Agency, 2020). Successfully coping with this enormous influx of refugees not only requires commitment through politics and authorities but also acceptance and support by the host population. Consequently, the question of how individuals in the receiving society react to increased immigration and might or might not support a successful integration of incoming refugees plays a growing role. Making a contribution to this debate, the present study focuses on host citizens' behavior that facilitates the well-being of citizens with a refugee background. More specifically, we investigate driving factors that promote host citizens' altruistic behavior toward refugees to provide empirically based insights for the development of policies for successful refugee helping and integration.

In Germany, we observe strong heterogeneity in behavior and attitudes toward refugees. On the one hand, there is a large number of people willing to support refugees. On the other hand, we observe growing support for extreme right-wing parties (Bock and Macdonald, 2019) being commonly accompanied by the perceived competition for resources and the general claim that welfare benefits should be restricted to natives (Cavaille and Ferwerda, 2016). At the same time, we observe an ever-growing gulf between rich and poor with an increasing number of social welfare recipients struggling to make a living. These diametrically opposed behavioral tendencies (direct help vs. direct harm) toward refugees reflect the risk of widening divisions in society. This is at the expense of the refugees as their fate is highly dependent on the host citizens' behavior and their willingness to facilitate integration. From the point of view of social justice, it is the majority group—the agent group—that have both the social responsibility and the (access to) resources required to help vulnerable groups in society (Goodman, 2011). As a consequence, it is the host citizens' behavior that provides the basis for successful integration as incoming refugees in many cases rely on their direct support. This support in the form of donations or personal assistance can be subsumed under the term “altruistic” behavior as it pursues the objective of improving the welfare of the recipient even though it is costly for the performer in terms of resources, time and energy (Fehr and Schmidt, 1999). The willingness to engage in altruistic behavior toward others is a fundamental issue for societies, which has been intensively studied over several decades (see Kogut and Ritov, 2017, for a review). Based on this important work on charitable giving in general, in the present paper we focus on factors that influence individuals' willingness to show altruistic behavior toward refugees in a real-world setting.

Given the immense importance of the host citizens' reaction toward immigrants in meeting the challenges posed by the refugee crisis, psychological research in this area has increased tremendously over the past few years (Jetten and Esses, 2018). However, the majority of the studies focuses either on general attitudes toward immigrants or specific attitudes toward immigrant helping and policies based on survey responses (e.g., Ceobanu and Escandell, 2010; Esses et al., 2017) being potentially limited in predicting actual behavior (Hilbig et al., 2014). Looking at host citizens' actual helping behavior toward refugees (that implies actual monetary consequences) we extend previous research in this field (see also Böhm et al., 2018, for a related approach).

In a pre-registered study, we aimed at extending our understanding of potential channels and drivers of altruistic behavior toward refugees in receiving societies. Based on the assumption that altruistic behavior is determined by multiple factors we consider various theoretical approaches in investigating the driving factors that promote altruistic behavior toward refugees. Primarily, we build on our findings from previous cross-cultural studies showing that altruistic giving toward others not only depends on (stable) characteristics of the giver (e.g., social preferences) but on characteristics of the receiver (e.g., national background) and their relation to each other (e.g., shared group membership) (Dorrough and Glöckner, 2016; Fiedler et al., 2018; Froehlich et al., 2021).

We generate an intergroup setting in which German student participants had the opportunity to engage in small acts of kindness toward individuals from their in-group (German students) vs. various out-groups, including groups with and without a refugee background. We use a set of different out-groups manipulating characteristics of the interaction to determine to what extent altruistic behavior is influenced by (1) shared local identity (i.e., individuals from the same city) and/or (2) shared student identity. We further assume two other main factors to be predictive for the extent of altruistic giving a person displays namely (3) perceived income differences between the self and the other (shared economic status) and (4) perceived closeness toward the other. Finally, we investigate further potential predictors of altruistic giving that refer to perceived receiver characteristics (perceived competition and perceived warmth) and orientations of the individual decision maker (i.e., their social and political orientation).

### Theoretical Predictions and Previous Findings Concerning Our Research Questions

#### Group Membership

Across various contexts it has been shown that shared group membership can increase peoples' tendency to behave altruistically toward others: Pedestrians are more likely to return a lost letter if the letter‘s owner is perceived to be a member of the same social category (Sole et al., 1975; Flippen et al., 1996); bystanders are more likely to help victims who are described as in-group as opposed to out-group members in emergency situations triggered by violent behavior (Levine et al., 2002) or natural disasters (Levine and Thompson, 2004; Cuddy et al., 2007; Kogut and Ritov, 2007). Players participating in social dilemma tasks are more willing to accept personal costs to benefit in-group compared to out-group members (De Cremer and Van Vugt, 1999; Goette et al., 2006; Simpson, 2006; Balliet et al., 2014). This phenomenon of in-group favoritism, that is the tendency to favor members of one's in-group over out-group members, can be explained by social identity theory and social categorization theory (Tajfel and Turner, 1979; Turner et al., 1987). Social identity theory assumes that the motivating principle underlying in-group favoritism is the need to attain and preserve a positive self-concept by maximizing the positive distinctiveness of the in-group in contrast to an out-group (Tajfel and Turner, 1979; Hewstone et al., 2002). This can be achieved by a preferential treatment of in-group compared to out-group members in form of a higher degree of altruistic behavior (see Balliet et al., 2014, for a meta-analysis). Extending this framework, self-categorization theory emphasizes the salience of shared group membership in social situations (Turner et al., 1987). When a social identity becomes salient, individuals perceive themselves as group members. As a consequence, intragroup similarities and intergroup differences are typically accentuated and in-group membership serves as a guide for perceptions, feelings, attitudes, intentions and behaviors (Brewer, 1991; Terry and Hogg, 1996).

One central characteristic of social identity is its dynamic nature as membership to a social group can be based on a wide range of objective and subjective criteria such as cultural background, socioeconomic status, or shared preferences etc. Consequently, individuals can be categorized on the basis of multiple social identities as they simultaneously belong to various social groups (Macrae et al., 1995; Rydell et al., 2009). Depending on the situational context of the interaction and salient self-categorization cues different social identities and corresponding self-concepts are activated. For instance, social labels can essentially determine self-categorization shaping attitudes and behaviors toward others. A study on multiculturalism indicated that non-minorities primed to think of themselves as European American (vs. White) were less racially prejudiced and more supportive of multiculturalism due to increased identification with ethnic minorities (Morrison and Chung, 2011).

Making similarities and shared characteristics of individuals salient has proven to be instrumental for increasing identification with others. Highlighting, for example, collective memories with others led to higher similarity ratings and subsequently to an in-group categorization of the respective persons (Tavani et al., 2017). When dissimilarities between in- and out-groups are made salient in-group favoritism is typically increased. For instance, Bilancini et al. (2020) showed that participants act more altruistically toward in-groups compared to out-group members when both groups differed in moral principles.

In the same vein, the perception of (dis)similarity appears to play a significant role in cross-cultural interactions. A recent study showed that perceived similarity promotes cross-cultural cooperation as individuals behave more pro-socially toward others from similar cultural groups (Froehlich et al., 2021).

Indicating that boundaries between groups are not fixed but rather constructed within individual decision situations, this evidence is in line with the common in-group identity model (Gaertner and Dovidio, 2000). The model emphasizes the social categorization process of recategorization, where former out-group members are perceived to belong to a superordinate common in-group and are consequently evaluated and treated more positively. Shared group identity can be induced by presenting factors that focus similarities between members of the superordinate group or by simply increasing the salience of existing shared group memberships (Dovidio et al., 2005). Studies that focus on the role of identity in determining attitudes toward immigrants and refugees indicate that it is particularly the conceptualization of in-group identity that determines responses to immigrants (Esses et al., 2001; Espinosa et al., 2018). These studies suggest that the more similar individuals perceive immigrants to be to the concept of what they as majority are and stand for, the more positive are their attitudes and behavioral tendencies toward immigrants (Jetten and Esses, 2018). For example, Espinosa et al. (2018) analyzed the role of identity overlap in attitudes toward immigrants and showed that promoting inclusive identities leads to more favorable attitudes. More specifically, they showed that the degree to which Sicilian teachers integrated their self-concept with immigrant students predicted positive attitudes toward these students. However, these positive effects did not generalize to immigration policy preferences so that it remains unclear if inclusive identities can have positive consequences for behavior toward immigrants.

In the present study we investigate the effect of a shared (vs. different) local identity, a shared (vs. not shared) student identity, and a shared (vs. different) economic status (i.e., perceived income differences) with refugees on the degree of altruistic behavior. We thereby selected three in principle unrelated criteria each of which might activate a shared social identity with refugees.

The first potentially shared identity refers to the place of residence with being residents of the same city representing a shared local identity. People often feel attached to the place they live in and develop a sense of belonging to a local community, which is based on a geographical territory (Tartaglia, 2006; Scannell and Gifford, 2010). The city of residence often carries emotional and symbolic significance for its residents and can be the basis for self-categorization. A person from Berlin may refer to himself/herself as a Berliner. Thus, the city of residence (in this case Berlin) is considered a social category whose group members are defined by shared living environment at the city level (Twigger-Ross and Uzzell, 1996). In line with predictions from social identity theory and social categorization theory, the local identification with the city and its residents facilitates “distinctiveness” from other cities (and its residents) and should promote a preferential treatment of local (same city) individuals.

H1: The magnitude of altruistic behavior increases with decreasing spatial distance since living in the same (vs. another) city increases the likelihood of activating a shared local identity. We respectively hypothesize that participants behave more altruistically toward others (e.g., refugees) living in the same (vs. another) city.

The second group categorization criterion for a shared identity refers to the social category of being a student, a shared student identity. Being a student is an important part of the self-concept for many university students (Gresham, 1995; Shields, 1995) as students invest a great amount of passion, time and financial resources in their academic education. Being a student defines their social status such as their position in society and brings certain economic benefits such as fiscal reliefs or discounted entry. In intergroup experiments, student identity is often used as a categorization criterion when a student in-group is contrasted with a non-student out-group (Sebby and Johnston, 2012). Based on predictions on social identity theory and social categorization theory, we expect a preferential treatment of other participants sharing a student identity.

H2: The magnitude of altruistic behavior increases when the receiving person is also a student (vs. not a student) due to the increased likelihood of an activation of a shared student identity. Hence, we expect that participants behave more altruistically toward students (e.g., refugee students) compared to non-students.

A third characteristic which may be used to evaluate others as being similar or dissimilar to the self is a shared economic status. In contrast to previous research on a shared local and student identity, research on shared economic status shows that perceived beneficial income differences between the self and another person are positively correlated with altruistic giving (Fehr and Schmidt, 1999). The literature shows that many people dislike unequal outcomes so that they behave altruistically toward others to avoid or reduce outcome-based inequality (e.g., Fehr and Schmidt, 1999; Bolton and Ockenfels, 2000; Tricomi et al., 2010). In the lab, altruistic behavior is typically measured by a Dictator Game where a dictator receives an endowment that he/she can split between himself/herself and an (unendowed) recipient (Engel, 2011). To test directly for inequality aversion, Korenok et al. (2012) changed the structure of the game. By increasing the recipient's endowment from 0 to an amount equal to the dictator's endowment, they showed that the amount given by the dictators reduced from 30% to <12% of the dictator's endowment. This result indicates that the inequality between dictator's and recipient's endowment is a key determinant of the dictator's giving.

In line with the assumption of inequality aversion and in contrast to H1 and H2, dissimilarity (given the other person is worse off) should trigger prosocial behavior. Cross-cultural studies give support for the assumption of inequality aversion showing that individuals make higher contributions to national out-groups that are financially worse off than their own nation (Tanaka and Camerer, 2013; Dorrough and Glöckner, 2016). In line with this, research on attitudes and behaviors in the context of refugee helping showed that the willingness to help refugees increased with their perceived neediness (Bansak et al., 2016; Böhm et al., 2018). Based on inequality aversion, we assume that the tendency to behave altruistically toward others depends on the individual's assessment of the other's financial position in relation to his or her own position. We measure perceived similarity between the own and the others' economic status (shared economic status) through the difference between own income and the believed income of the respective out-group member.

H3: The magnitude of altruistic behavior increases with increasing income differences between the self and the other person. Hence, we expect that participants' willingness to give money toward others (e.g., refugees) increases with the degree to which they perceive the other to be financially worse off compared to themselves (as measured by perceived income differences).

Perceived similarities are the precursor of shared identity (Yamagishi and Kiyonari, 2000). Hence, to create shared identities, similarities between groups are often made salient. However, focusing on similarities can also increase perceived competition and reduce prosocial behavior if similarities are not sufficient for generating a shared identity to include others into a perceived in-group. In such cases, perceived intergroup competition is particularly increased when the out-group is similar to the in-group on dimensions relevant (e.g., skills) to obtaining resources (Esses et al., 1998). It is particularly important to account for triggers of increased competition because competition can lead to increased conflict between groups in form of prejudice and negative behavior toward the out-group (Sherif et al., 1961; Jackson, 2002). In line with this argument, a study on attitudes toward immigrants indicated that making similarities on work-related traits between the in-group and an immigrant out-group salient led to increased perceived competition such as more prejudice and more negative attitudes toward immigrants (Zarate et al., 2004). Accordingly, it seems appropriate to take into account the possibility that perceiving others as similar to the self does not necessarily lead to an activation of common in-group membership but rather increases perceived competition and thus having a negative effect on prosociality. Following this line of reasoning, being a student and living in the same city might go along with an increase in perceived competition for academic and local resources, which could result in a decrease in altruistic giving toward students (vs. non-students) and locals (vs. non-locals).

#### Perceived Closeness

One further measure that can be expected to predict altruistic behavior by capturing differences concerning whether shared identity is activated or not is perceived closeness. The recognition of shared group membership typically strengthens the perceived psychological connection between the self and in-group members with individuals expanding their self-concepts to include identities of their in-groups (Aron et al., 1992). However, individuals differ in the degree to which they include the in-group in the representation of the self. While some individuals perceive a high degree of closeness to the in-group and its members experiencing a high self-in-group overlap, others do so to a much smaller extent (Tropp and Wright, 2001). The perception of social closeness is not restricted to in-groups as individuals can also perceive out-groups (and its members) as socially close including them in their self-concept (self-out-group overlap). When an intergroup context is salient, however, the perceived self-in-group overlap is higher than the perceived self-out-group overlap (Schubert and Otten, 2002). Consequently, the activation of a shared social identity should lead to an increase in perceived closeness with similar others (that share a local or student identity with the self) being perceived as closer compared to dissimilar others (non-locals or non-students).

Moreover, perception of social closeness is per se highly relevant for altruistic behavior toward others. In a cross-national study, we showed that the effect of perceived social closeness on altruistic behavior toward fellow countrymen and persons from other nations goes beyond the mere dichotomous difference between in- and out-group having additional predictive value (Fiedler et al., 2018). Generally, as one would intuitively expect, a high (low) degree of perceived social closeness toward another person is associated with increased (decreased) altruistic behavior (e.g., Aron et al., 1991; Hoffman et al., 1996; Burnham, 2003; Rachlin and Jones, 2008). Initial studies on refugee helping point in the same direction indicating that a high degree of perceived closeness measured as the inclusion of the self in the refugee out-group is associated with higher levels of altruistic behavior (Nyeste, 2017). Following from this, we account for individual differences in the perception of closeness toward individuals from different groups, which we assume to predict altruistic giving.

H4: The magnitude of altruistic behavior increases with increasing perceived self-other overlap. Hence, we expect that participants behave more altruistically toward others (e.g., refugees) they perceive high degrees of self-other overlap with.

The perception of closeness is typically increased when a shared identity becomes salient (Schubert and Otten, 2002). As perceived social closeness strongly predicts altruistic behavior (e.g., Aron et al., 1991; Hoffman et al., 1996; Burnham, 2003; Rachlin and Jones, 2008), we predict that perceived closeness mediates the effect of our main predictors (shared local identity, shared student identity, perceived income differences) on altruistic giving.

H4a-c: The effect of our main predictors shared local identity (H4a), a shared student identity (H4b) as well as perceived income differences (H4c) on altruistic behavior is mediated by perceived closeness. Hence, we expect that similar others that share a local/student identity with the self and are evaluated as similar concerning their economic status (i.e., have only small income differences) are perceived as socially closer, which also mediates the effects of these factors on altruistic behavior.

#### Further Predictors of Altruistic Giving

In addition to the above presented main predictors for altruistic giving we assessed further influencing factors, that refer to characteristics of the receiver (perceived competition and perceived warmth) on the one hand and to individual orientations of the individual decision maker (i.e., his or her social and political orientation) on the other hand.

##### Receiver Characteristics: Perceived Competition and Warmth

As introduced above, perceived competition can increase antisocial behavior in the intergroup setting (Sherif et al., 1961). According to realistic group conflict theory (RGCT), direct competition for access to limited resources can lead to intergroup bias and conflict between groups in form of prejudice and hostile behavior toward the out-group (Sherif et al., 1961; Jackson, 2002). It is assumed that perceived group competition is likely to take the form of a zero-sum contest over resources (believing that the more one group obtains, the less is available to the other group) that is accompanied by high degrees of perceived threat (Sherif and Sherif, 1953). It is important to note that it is the (subjective) perception of competition over resources rather than actual competition that affects one's attitude toward the other group leading to group conflict (Esses et al., 1998). In line with prediction from RGCT, it has been shown that perceived intergroup competition decreases altruistic and cooperative behavior toward out-group members in both adults (e.g., Platow et al., 1999; Bornstein et al., 2002; Bauer et al., 2014) and children (e.g., Rhodes and Brickman, 2011; Abrams et al., 2015). RGCT is frequently used to explain anti-immigrant attitudes as numerous studies indicate that negative attitudes toward immigrants are often a result of the host society perceiving competition toward immigrants (e.g., Quillian, 1995; Scheepers et al., 2002; Semyonov et al., 2004, 2006; Meuleman et al., 2009; Gorodzeisky, 2011). Arguably, opponents of refugee integration might perceive refugees as rivals in competition for resources, such as jobs, housing, and other goods to which they feel having first claim on. With regard to helping behavior toward refugees, RGCT would accordingly predict that increased competition over (limited) resources leads to negative behavior (e.g., direct harm) or the absence of positive behavior (e.g., reluctance to help) toward refugees.

H5: The magnitude of altruistic behavior decreases with increasing perceived competition. Hence, we expect participants to behave less altruistically toward others (e.g., refugees) they perceive a high degree of competition toward.

A further receiver characteristic that can be expected to influence the degree of altruistic behavior is the perception of the other person on the stereotype dimension of warmth/communion. Representing effective tools to facilitate information processing and response generation (e.g., Bodenhausen and Lichtenstein, 1987; Fiske and Neuberg, 1990) stereotypes correspond to perceivers' beliefs about characteristics that define a group and its members. Stereotypes generally provide information about what typical group members are like, think, feel and do (Fiske and Pavelchak, 1986; Gilbert and Hixon, 1991; Macrae et al., 1995). This allows individuals to make predictions about group members' behavior (Hamilton et al., 1990) being able to adapt their own judgments, decisions and behaviors accordingly (Wheeler and Petty, 2001). For cross-cultural interactions, stereotypes have crucial importance as there are culturally shared stereotypes about national groups that predict intergroup attitudes and behavior (Cuddy et al., 2008). Stereotype content models (Fiske et al., 2002; Koch et al., 2016) propose that stereotypes vary on the dimensions of agency/competence, communion/warmth, and one model additionally assumes conservative/progressive beliefs as third dimension (Koch et al., 2016). The perception of warmth/communion is based on whether the other's intentions are perceived as friendly and has been identified to predict the willingness to help others. Out-groups perceived as warm elicit active facilitation such as helping behavior, whereas out-groups being stereotyped as cold attenuate active harm (Cuddy et al., 2008; Sanchez and Bonam, 2009; Becker and Asbrock, 2012). In line with this, a recent study found warmth/communion perception to be positively (negatively) correlated with facilitation (harm) behavior toward asylum seekers (Bye, 2020). Consequently, perceiving the other as warm should influence altruistic giving (toward refugees).

H6: The magnitude of altruistic behavior increases with higher perceived values on the warmth/communion dimension. Hence, we expect participants to behave more altruistically toward others (e.g., refugees) they perceive as warmer (as compared to less warm).

##### Social and Political Orientations of the Individual Decision Maker

The degree of altruistic behavior shown toward others (e.g., toward refugees) can be further influenced by characteristics of the individual decision maker. One important factor in this regard is the individual's general (pro)social orientation. While some individuals tend to act out of pure self-interest, others do care about the well-being of their fellow people sacrificing own resources to help others (see e.g., Fehr and Schmidt, 1999; Van Lange, 1999; Balliet et al., 2009). Prosocial preferences have proven to be fairly stable over long periods of time and contexts (Carlsson et al., 2014; Murphy and Ackermann, 2014) and represent a valid predictor for individual's altruistic behavior (e.g., Fischbacher et al., 2001; see also Balliet et al., 2009 for a meta-analysis). Evidence on the interplay between prosocial preferences and intergroup context suggests that individuals with a general prosocial orientation give relatively less to out-groups than individuals with a general pro-self orientation (De Dreu, 2010; Abbink et al., 2012; De Dreu et al., 2015). There is, however, diverging evidence (see e.g., Thielmann and Böhm, 2016) that calls this result into question. In a cross-cultural study on in-group favoritism, we found that altruistic giving to the cultural in-group significantly predicted altruistic giving to cultural out-groups (Fiedler et al., 2018). In the context of refugee helping, it has been shown that differences in general prosocial orientation determine helping intents as higher degrees of prosocial orientation were associated with more helping behavior toward refugees. This provides evidence for the theoretical assumption that prosociality is rather universal and not bounded by group membership (Böhm et al., 2018). To further validate this finding, we investigate if individuals' general prosocial orientation measured in form of altruistic giving toward in-group members (local German students) is predictive for altruistic giving toward others (e.g., refugees).

H7: The magnitude of altruistic behavior to persons from other groups increases with altruistic behavior to the in-group (i.e., students from the same city). Hence, we expect participants that are more altruistic toward local German students to behave also more altruistically toward others (e.g., refugees).

Individuals' political orientation was shown to be associated with helping behavior, especially in the context of refugee helping. This might be partially due to the fact that political orientation is related to social orientation (Balliet et al., 2018). Political orientation is typically measured on a left (liberal) to right (conservative) continuum (e.g., Cavazza and Mucchi-Faina, 2008; Jost et al., 2009). While left-wingers often support equality among individuals pursuing more prosocial goals, right-wingers tend to accept hierarchies and inequalities between individuals and groups and pursue more pro-self goals (Thorisdottir et al., 2007; Carney et al., 2008; Sheldon and Nichols, 2009). Note, however, that there is also empirical evidence indicating differences between the effects of political orientation and prosocial orientation in that prosocial orientation sometimes also is related to giving less to out-groups (De Dreu, 2010; Abbink et al., 2012; De Dreu et al., 2015). The general positive association between political left-wing orientation and altruism (Zettler and Hilbig, 2010) is particularly relevant with regard to immigrant integration as extreme right-wing voting is strongly associated with anti-immigration sentiments (Rooduijn et al., 2017; Holbrook et al., 2018) and increased (decreased) anxiety (self-efficacy) about the consequences of the “refugee crisis” (van Prooijen et al., 2018). Right-wing parties typically take advantage of individuals' future anxieties about not being able to compete mobilizing irrational fears of foreigners in general and refugees in particular (Beland, 2020). The fear that immigration represents a threat to the development of the host society's economy, culture and safety is directly associated with negative attitudes (Jedinger and Eisentraut, 2020) and reduced helping behavior toward refugees and immigrants (Burhan and Leeuwen, 2016). In a similar manner, nationalism has been shown to be linked to prejudice against and discriminatory behavior toward immigrants (e.g., Billiet et al., 2003; Wagner et al., 2010). With regard to altruistic behavior toward refugees, recent studies indicate that political left-wing orientation goes along with helping intents toward refugees (Bajrami et al., 2017; Böhm et al., 2018). Within the present study, we will further explore this influence of political left-wing orientation on altruistic behavior toward others (e.g., refugees).

H8: The magnitude of altruistic behavior increases with political left-wing orientation. Hence, we expect participants holding a political left-wing (vs. right-wing) orientation to behave more altruistically toward others (e.g., refugees).

## Method

The study was pre-registered^1^ (see https://osf.io/p8ce6) and performed in accordance with the DecisionLab's regulations (no deception, full incentivization). Informed consent was obtained from all participants. All measures, manipulations, and exclusions in the study are reported completely and transparently. Sample size was determined before data collection based on a pragmatic reason that the study was run in a battery with an unrelated study. Note that a *post-hoc* sensitivity analysis^2^ using G*power (Faul et al., 2009) revealed that the sample size used in this study allows for the detection of small to medium effects (*f* = 0.18/ *d* = 0.36) with a power (1-β) = 0.95. The analysis was only run after all responses were collected. The complete instructions (https://osf.io/e9a6x/) and data (https://osf.io/hxar5/, including analysis script) are available at the Open Science Framework.

### Participants and Design

We applied a 2 [within: shared local identity (same city) vs. different local identity (other city)] × 3 [between: shared student identity (refugee student) vs. different social identity (refugee, social welfare recipient)] mixed factorial design to investigate potential drivers of altruistic behavior. One hundred ninety-six undergraduates^3^ of the University of Bonn, Germany (59% female; 18–32 years of age, M = 21.09, SD = 2.70) took part in the study. Being mainly first-year students recruited from the DecisionLab subject pool via the database system ORSEE (Greiner, 2015), participants had no prior exposure to economic experiments. The experiment was part of a two-study battery and lasted about 25 min including an online part (10 min) and a lab part (15 min). In addition to their payment for the first study which was not related to the study reported here, participants were compensated with a show-up fee of 4.00€ plus an incentivized bonus payment of 0 to 2.00€ which was contingent on their donation behavior. Resulting earnings ranged from 4.00€ to 6.00€ (average payment 5.33€).

### Materials and Procedure

At least 12 h before coming to the lab participants completed a pre-questionnaire, which was administered online via unipark (www.unipark.de). This online questionnaire included personality factors namely left-wing orientation, nationalism, and future anxiety assumed to be relevant for altruistic giving. We determined participants' political orientation using the Left-Right Self-Placement (Breyer, 2015), which is frequently used in political and social surveys. Thereby participants placed their own political orientation on a 11-point scale from left to right (i.e., single item measure). In addition to political left-wing orientation we measured nationalism with two items from the nationalism and constructive patriotism scale (Davidov, 2009, “The world would be a better place if people from other countries were more like the Germans,” “Generally speaking, Germany is a better country than most other countries”; α = 0.67). Future anxiety was assessed by 12 items referring to different life domains such as work, academic education, children (e.g., “I am afraid that I am not going to find a workplace after I finish my studies”). Besides these personality factors participants were asked to indicate their beliefs about the monthly net income of members from different social groups on the basis of 21 categories ranging from 250€ to 5,000€. The list of social groups included the out-groups participants were later presented with in the lab part of the study (e.g., students, refugees, student refugees, welfare recipients) but also further groups as distractors (e.g., hairdresser, medical doctor). Since the demographic questions in the end of the questionnaire included an information about participants own net income, we were able to calculate perceived income differences. Within our study perceived income differences functioned as a proxy for similarity in economic status.

**Figure 1:**
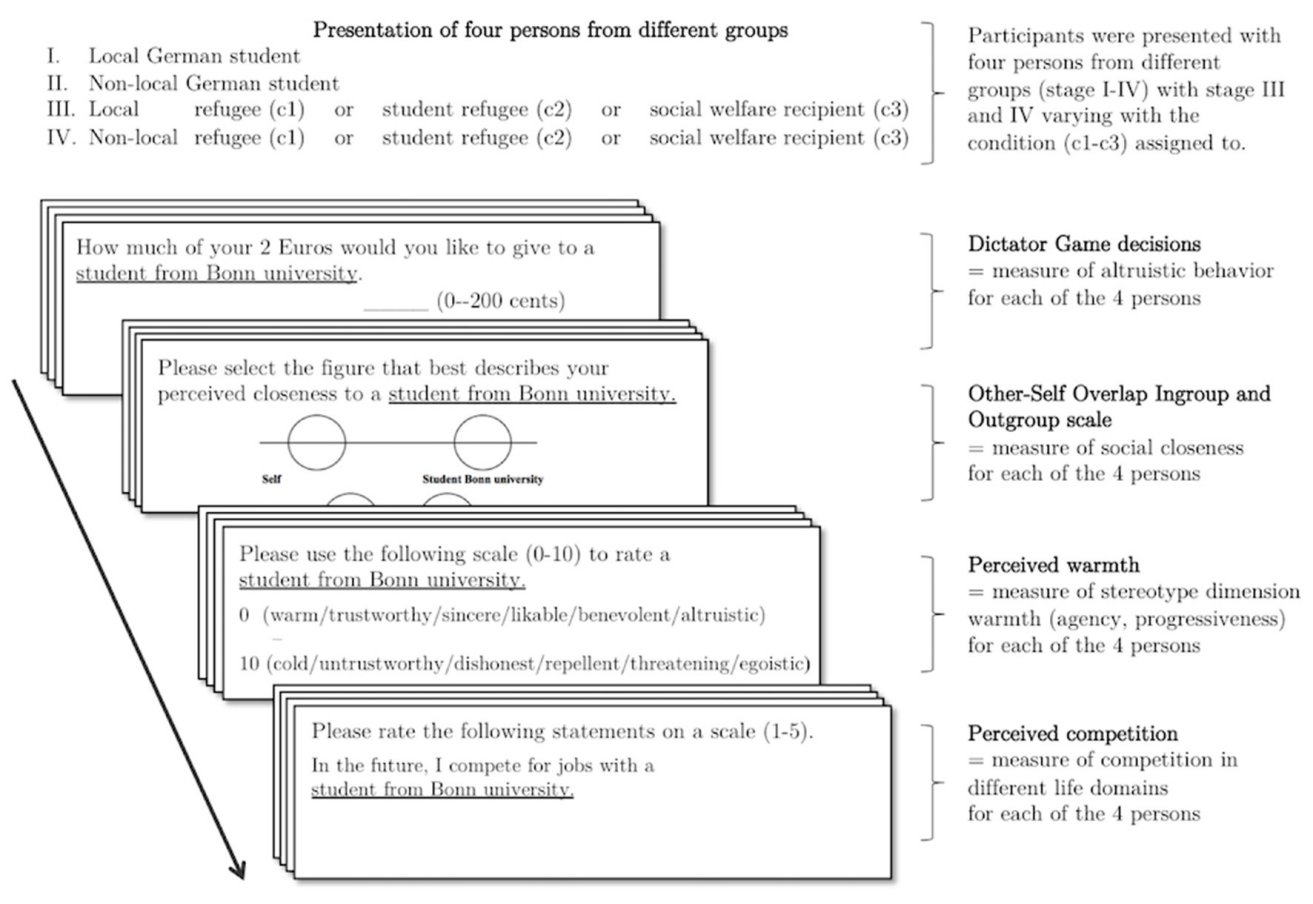
Experimental procedure.

Within the lab experiment part, each participant was presented with four persons from different groups with the group membership being the only information provided about these four persons. An overview of the procedure including the individual materials is provided in Figure 1. In stages one and two, all participants were presented with a (local) German student from the University of Bonn (local and student in-group) and a German (non-local) student from another city in Germany.^4^ The out-group members presented subsequently varied with the condition, which participants were randomly assigned to on their arrival at the lab. Thus, in stage three and four, participants were matched with a local and a non-local refugee (refugee condition) or a local and a non-local student refugee (student refugee condition) or a local and a non-local social welfare recipient (social welfare recipient condition) as between-subject factor. The latter receiver group served as a German (same nationality) comparison group since social welfare recipient share relevant characteristics with the refugees' population in terms of social and economic status.

During the experiment participants had the chance to donate -suited to the season- little presents (at costs of up to 2€) to the four persons matched with and were asked to evaluate them regarding different characteristics. The same ordering of the presented persons was applied for all tasks. The core dependent measure altruistic giving was assessed using a Dictator Game. The core predictor perceived closeness was measured using the Overlap of Self-In-group (OSI) and Overlap of Self-Out-group (OSO) Scales (Aron et al., 1992). The order of both measures was counterbalanced. As further predictors we included perception of stereotype contents (e.g., warmth/communion) and competition regarding the other person. To control for the degree of personal contact with people from the respective out-groups, participants answered three contact questions at the end of the study, namely whether they had ever encountered, had regular contact with persons from the respective groups or whether they had family or close friends from these groups.

#### Altruistic Giving

We measured altruistic giving in form of small acts of kindness using simple one-shot Dictator Games. Participants received an endowment of 2.00€ which they could split between the self (the dictator) and the other person (the receiver) in steps of 1 cent. Participants made four Dictator Game decisions for each of the four persons matched with. Participants were informed that one of their decisions would be randomly selected to be relevant for their payment whereby the amount given to the self would be paid out in cash and the amount given to the other person would be paid out in form of a little present.

#### Perceived Closeness

As a subjective measure of social closeness toward members of the different groups, participants assessed the Overlap of Self-In-group (OSI) and the Overlap of Self-Out-group (OSO) Scales, which are often employed in research on intergroup relations (Aron et al., 1992). Out of seven pictures of two increasingly overlapping circles, one representing themselves and the other representing the respective group, participants were asked to select the figure that best described their perceived closeness to in- (OSI) and out-group members (OSO). The order of the presented in- and out-group members was the same as in the Dictator Game.

#### Perceived Warmth/Communion

Participants evaluated each of the four persons matched with on the stereotype dimensions of warmth/communion derived from the 2D ABC model of stereotype content about social groups (Koch et al., 2016). Participants were asked to rate the other person on a scale ranging from 0 (untrustworthy/dishonest/repellent/threatening/cold/egoistic) to 10 (trustworthy/sincere/likable/benevolent/warm/altruistic). Additionally, we assessed the other two dimensions agency/socioeconomic success (powerless-powerful, poor-wealthy, low status-high status, dominated-dominating, unconfident-confident, and unassertive-competitive) and conservative-progressive beliefs (traditional-modern, religious-science-oriented, conventional-alternative, and conservative-liberal).

#### Perceived Competition

Participants indicated the degree of perceived competition with the other person regarding 12 items from different life domains such as work, academic education, children (child benefit, childcare place) governmental support such as pension and unemployment benefit, access to affordable housing and public resources such as parking or public transport. Items were presented as statements for which participants were asked to indicate their agreement on five-point Likert scales (e.g., “I compete for jobs with students from the university of Bonn”; 5 = very much, 1 = not at all). In order to have a general competition measure an average score for perceived competition was calculated based on the 12 individual competition measurements.

## Results

### Main Predictors of Altruistic Giving

**Figure 2:**
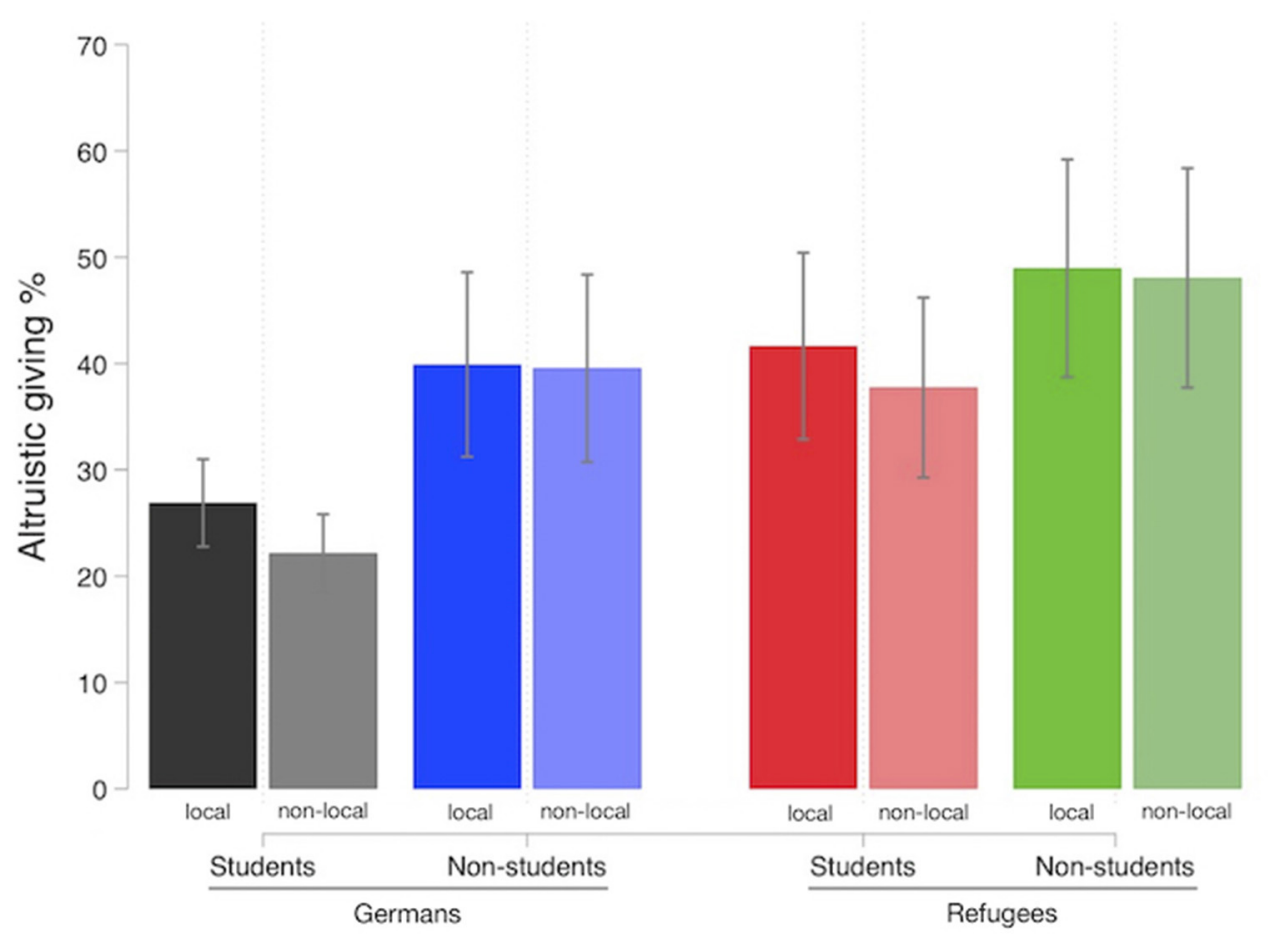
Altruistic giving (in percentages) for all groups of receivers: German students, non-student Germans (= social welfare recipients), refugee students and non-student refugees. Bars in saturated colors represent the receivers with a shared local identity whereas bars in brighter colors represent non-local receiver groups. Error bars represent 95% confidence intervals.

Figure 2 presents altruistic giving toward all groups of receivers. We observed markedly different degrees of altruistic giving toward the various groups. Descriptively, refugees received more money than Germans, students received less than non-students and locals (people living in the same city) received more money than non-locals (people living in another German city). Still, the descriptive results have to be interpreted cautiously since they do not control for systematic differences between groups concerning hypothesized relevant factors such as their perceived income (and others).

To account for these simultaneous influences that are inherent in our design, we test the directed pre-registered hypotheses (H1–H8) concerning the factors influencing altruistic giving simultaneously using a regression approach and one-sided tests for each predictor. To account for the repeated measurement design (i.e., participants indicated how much money they would give to various groups) we thereby applied mixed effects models with random person intercepts. To test hypotheses H1–H4, we regressed altruistic behavior on our four main predictors controlling for the remaining manipulated effects by including group dummies (i.e., the main analyses included dummies for local identity, student identity, refugee and a refugee*student interaction) using welfare recipients as control group (i.e., comparisons against non-local welfare recipients). This allowed us to simultaneously estimate the unique contributions of each effect while controlling for the remaining effects (i.e., partial correlations after controlling for all other effects) that are relevant for our hypotheses. In a first step, we included the predictors shared local identity (i.e., residents from the same or another German city) and shared student identity (i.e., student or other social group) as well as the control factors refugee and refugee*student in the regression (see Table 1, column 1). We then added perceived income differences (see Table 1, column 2), and closeness (i.e., OSI & OSO, see Table 1, column 3) separately into the model. Finally, we included all four main predictors simultaneously (Table 1, column 4). As a robustness check, we rerun the model two times (1) controlling for all interaction terms of the predictors and group dummies (note that results do not qualitatively change in this analysis) and (2) excluding German students to use (pro)social orientation (measured as in-group giving) as a control variable (as pre-registered). Note that our analyses differ from the pre-registration regarding the inclusion of appropriate control variables (e.g., group dummies), which we missed to pre-register but subsequently added to the analyses for logical reasons following the suggestion of an anonymous reviewer. Results do not change qualitatively when running the pre-registered analysis except for the effect of perceived closeness (H4) and perceived competition (H5) where the missing control factors lead to effects in the opposite direction (see Supplementary Material for a detailed report of the results of the pre-registered analyses).

**Table 1:** Altruistic giving predicted by the main predictors shared local identity and shared student identity (column 1), perceived income differences (column 2) and closeness (column 3) and all main predictors (column 4) for the overall sample.

	**(1)**	**(2)**	**(3)**	**(4)**
**Predictors**
Shared local	6.45^*^	6.45^*^	4.75	4.92^*^
Identity (*yes = 1*)	(2.30)	(2.33)	(1.63)	(1.71)
Shared student	−22.20^[***]^	−23.62^[***]^	−26.28^[***]^	−27.22^[***]^
Identity (*yes = 1*)	(−5.85)	(−6.25)	(−6.21)	(−6.49)
Perceived		0.04^***^		0.04^**^
Income diff		(3.36)		(3.23)
Perceived			2.81^*^	2.53^*^
Closeness			(2.17)	(1.96)
**Controls**
Refugee	31.20^***^	26.81^***^	33.19^***^	28.78^***^
	(8.21)	(6.73)	(8.51)	(7.02)
RefugeeX	5.57	8.59	10.09	12.53
Student identity	(0.70)	(1.08)	(1.22)	(1.53)
Constant	67.51^***^	67.68^***^	67.76^***^	67.90^***^
	(16.88)	(16.75)	(17.00)	(16.86)
Observations	784	784	784	784

Numbers refer to unstandardized regression coefficients for the predictor variables (all predictor variables were centered for the analysis and before calculating the interaction term refugee*shared student identity), z statistics in parentheses.

* *p < 0.05*,

** *p < 0.005*,

*** *p < 0.001, all stars indicate one-sided test results, if effects are in the opposite direction to the predicted hypotheses stars are reported in brackets “[]” for informative reasons only*.

In addition to the pre-registered analysis of the overall sample (see Table 1), we categorized receivers based on their nationality in a refugee (student and non-student refugees, see Table 2, column 1) and a German^5^ (German students and social welfare recipients, see Table 2, column 2) subsample and further provided a more detailed individual subgroup analysis (see Table 2, column 3–6).

**Table 2:** Subgroup analyses of altruistic giving for the refugee (student and non-student refugees, column 1) and German (German students and social welfare recipients, column 2) subsample and for each individual receiver group separately (columns 3–6).

	**Refugees**	**Germans**	**German students**	**Student refugees**	**Non-student refugee**	**Welfare recipients**
**Predictors**
Shared	4.13^*^	6.54^*^	6.82^**^	6.90	1.75	0.69
Local identity	(1.86)	(2.14)	(2.89)	(1.61)	(1.28)	(0.19)
(*yes = 1*)						
Shared student	−19.22^[*]^	−27.74^[***]^				
Identity	(−1.74)	(−5.81)				
(*yes = 1*)						
Perceived	−0.01	0.03^**^	0.02	−0.03	−0.01	0.01
Income diff	(−0.52)	(2.79)	(0.96)	(−0.74)	(−0.13)	(0.49)
Perceived	4.07^*^	0.89	2.40^*^	2.98	3.66^**^	1.43
Closeness	(2.26)	(0.66)	(2.09)	(0.98)	(3.02)	(0.56)
**Controls**
Prosocial	0.68^***^			0.65^***^	0.70^***^	0.56^***^
Orientation	(7.14)			(5.49)	(4.75)	(4.34)
(In-group giving)						
Constant	53.05^***^	69.77^***^	36.17^***^	37.70^**^	52.93^***^	43.20^***^
	(5.11)	(12.46)	(6.60)	(3.16)	(4.23)	(3.57)
Observations	262	522	392	130	132	130

*Numbers refer to unstandardized regression coefficients for the predictor variables, z statistics in parentheses*,

* *p < 0.05*,

** *p < 0.005*,

*** *p < 0.001, all stars indicate one-sided test results, if effects are in the opposite direction to the predicted hypotheses stars are reported in brackets “[]” for informative reasons only*.

The overall analysis revealed a significant effect for local identity on DG giving (see Table 1 and Figure 2). In line with H1, there was a unique effect of local identity in that participants' altruistic giving increased for people living in the same city as compared to people living in another city. This effect holds for the subsamples of refugee and German receivers (see Table 2). No significant difference in magnitude was observed between the two subsamples indicated by a non-significant interaction of local identity and being a refugee in a further analysis (see Supplementary Table 1a). Looking at each receiver group individually, we observe this effect to be significant for German students and a not significant trend for student refugees and non-student refugees with locals receiving substantially more allocations than non-locals (see Table 2). Interestingly, this is not true for the receiver group of social welfare recipients as local social welfare recipients do not receive more money compared to non-local social welfare recipients.

There was no support for our second hypothesis in that—at least in this context—a shared student identity did not increase but significantly decrease altruistic giving as compared to non-student groups in the overall sample (see Table 1). We also observed this significant negative effect of a shared student identity on altruistic giving for the subsamples of refugees and Germans (see Table 2) with this effect being similar in magnitude (see Supplementary Table 1a). Hence, participants allocated significantly less money toward receivers they share a student identity with (vs. not). The unexpected opposite effect of shared student identity on altruistic giving (despite controlling for income differences) might be due to increased perceived competition toward this highly similar out-group, which we explore in more detail below.

Regarding our third main predictor, perceived income differences (shared economic status), the analysis of the overall sample revealed a significant effect of perceived income differences on altruistic giving supporting H3 (see Table 1, column 2). As expected, participants allocated more money to others, they perceived to be financially worse off compared to themselves. However, rerunning the analysis additionally controlling for in-group giving (German students are naturally excluded in this analysis) the effect of perceived income differences is no longer significant (see Supplementary Table 1b). In line with this, we only find this effect for German receivers (where controlling for in-group is not possible) but not for refugee receivers (see Table 2).

Investigating the influence of a shared identity on perceived closeness, the analysis showed, as assumed, that participants differentiated between the receiver groups (see Supplementary Table 2). Specifically, they reported feeling significantly closer toward student receivers compared to non-student receivers. This effect of students being perceived as significantly closer than non-students holds for both the refugee and the German subsample. Similarly, we observe a significant effect of a shared local identity on perceived closeness. This effect holds for both student receiver groups (German students and refugee students) as participants perceived significantly more closeness toward local (vs. non-local) student refugees and local (vs. non-local) German students. Contrary to what we would have expected, participants further reported to feel closer when facing receivers with less vs. similar or even more income. Note, however, that this overall significant effect was mainly driven by the subsample of social welfare recipients since there was no such effect for the other receiver groups.

Testing H4, we observed a significant effect of perceived closeness on altruistic giving in the overall (see Table 1, column 3) as well as the refugee receiver sample (see Table 2, column 1) and no refugee*closeness interaction (see Supplementary Table 1a). Simply put, the closer participants feel toward (refugee) receivers, the more altruistic they behave toward them. In line with the previously reported findings, this does not apply for the German subsample (German students and social welfare recipients) as German students are perceived as closer than social welfare recipients while the former receive less than the latter. Contrasting non-student refugee receivers with the German comparison group of social welfare recipients, we see that perceived closeness strongly predicts altruistic giving toward non-student refugees (*b* = 3.66, *z* = 3.02, *p* < 0.001), while it has no impact on giving toward social welfare recipients (*b* = 1.43, *z* = 0.56, *p* = 0.572).

To test our hypotheses 4a−4c we investigated whether effects of local identity, social identity and income inequality on altruistic giving are mediated by differences in perceived closeness between groups using multi-level mediation analyses with bootstrapped standard errors (Krull and MacKinnon, 2001).^6^ When adding perceived closeness to the regression predicting altruistic giving, the effect of a shared local identity was no longer significant (see Table 1, column 3) indicating a potential partial mediation effect of perceived closeness as predicted in H4a. Indeed, the mediation analysis revealed a significant indirect effect (one-sided) for a shared local identity in the overall sample (coeff = 1.5, z = 1.73, *p* = 0.08 (two-sided); bias-corrected CI_0.95_ (−0.21, 3.26); 24% of the total effect are mediated; see Supplementary Figure 1a). However, when rerunning the mediation analysis excluding German students and controlling for in-group giving the effect was no longer significant (bias-corrected CI_0.95_(−0.29, 0.99), see Supplementary Figure 1a). Also for the refugee subsample there was no significant indirect effect for a shared local identity [bias corrected CI_0.95_(−0.44, 1.74), see Supplementary Figure 1b]. Hence, the mediation effect seems to be small or not overly robust. Since we found significant main effects for a shared student identity on altruistic giving and for perceived income differences on perceived closeness in the opposite direction than expected the requirements for mediation analyses (e.g., Baron and Kenny, 1986) were not met (rejecting H4b and H4c).

### Further Predictors of Altruistic Giving

The remaining assumed predictors of altruistic giving concerning perceived receiver characteristics (H5 and H6) and individual characteristics of the giving person (H7 and H8) were investigated again using a simultaneous regression model for which the results are presented in Table 3. Contrary to H5, perceived competition did not influence altruistic giving, neither in the overall sample nor when restricting the analysis to individual receiver groups (see Table 3). In the overall sample, there was support for H6 in that altruistic giving increased, the more warm-hearted receivers were perceived. Interestingly, this effect of perceived warmth on giving only holds for refugee but not for German receivers (see Table 3 and Supplementary Table 3 for the significant interaction of warmth and being a refugee).

**Table 3:** Altruistic giving predicted by perceived competition, perceived warmth, political left-wing orientation and prosocial orientation for the overall (all receivers, column 1), refugee (student and non-student refugees, column 2) and German (German students and social welfare recipients) sample and for each individual receiver group separately (column 4–7).

	**Overall**	**Refugees**	**Germans**	**German students**	**Student refugees**	**Non-student refugee**	**Welfare recipients**
**Predictors**
Perceived	1.56	5.15	2.81	4.10	4.64	−0.78	2.06
Competition	(0.43)	(0.98)	(0.71)	(1.00)	(0.60)	(−0.17)	(0.28)
Perceived	6.02^***^	10.97^***^	0.49	1.65	7.42^+^	6.70^**^	0.96
Warmth	(3.68)	(3.96)	(0.27)	(0.81)	(1.64)	(2.70)	(0.20)
Political	−6.81^**^	−12.23^***^	−4.33^*^	−3.76	−10.78^*^	−13.62^**^	−4.61
Orientation	(−2.85)	(−4.01)	(−1.84)	(−1.58)	(−2.32)	(−3.19)	(−0.93)
(0 = left)							
Prosocial		0.67^***^			0.66^***^	0.66^***^	0.55^***^
Orientation		(7.88)			(5.88)	(4.97)	(4.25)
(In-group giving)							
**Controls**
Shared	5.61^*^	3.35	6.77^*^	8.59^***^	6.06	2.00	0.31
Local identity	(1.98)	(1.44)	(2.32)	(4.05)	(1.40)	(1.28)	(0.08)
(*yes = 1*)							
Shared student	−29.41^[***]^	−26.37^[*]^	−29.29^[***]^				
Identity	(−6.15)	(−2.57)	(−5.29)				
(*yes = 1*)							
Refugee	30.30^***^						
(*yes = 1*)	(8.00)						
Refugee*shared	9.90						
student identity	(1.22)						
Perceived		0.02	0.04^**^	0.02	−0.01	0.04	0.01
Income diff		(0.72)	(3.01)	(1.32)	(−0.27)	(1.02)	(0.52)
Observations	784	262	522	392	130	132	130

*Numbers refer to unstandardized regression coefficients for the predictor variables (only for the overall sample, regression coefficients are centered), z statistics in parentheses*,

+ *p < 0.1*,

* *p < 0.05*,

** *p < 0.005*,

*** *p < 0.001, all stars indicate one-sided test results. If effects are in the opposite direction to the predicted hypotheses stars are reported in brackets “[]” for informative reasons only. Due to the fact the analyses include subsamples that are split up by relevant factors and the not fully crossed design, the analyses include different control variables*.

With regard to individual orientations, we find support for H7 and H8 in the overall sample. For prosocial orientation (H7), we conducted a separate regression analysis since German students need to be naturally excluded in this analysis. This overall analysis revealed a significant and strong effect of prosocial orientation on altruistic giving (*b* = 0.63, *z* = 8.23, *p* < 0.001) that holds when controlling for perceived income differences, group dummies and their interactions with prosocial orientation (*b* = 0.64, *z* = 8.42, *p* < 0.001). Also political orientation was shown to be a robust predictor for altruistic giving in the overall sample (see Table 3). The more prosocial their orientation (high degrees of altruistic giving toward their in-group) and the more left-wing their political orientation, the more altruistic participants behave toward others.

Both effects hold for the individual subgroups of receivers (see Table 3). Note that the effect of political left-wing orientation on altruistic giving toward refugees is stronger than when predicting altruistic giving toward German receivers (see interaction effect, Supplementary Table 3). For German social welfare recipients, political orientation had no significant predictive power for giving behavior. For decision makers' prosocial orientation, we observe a similar pattern of results for German social welfare and non-student refugee receivers (no interaction effect, see Supplementary Table 3). In Supplementary Table 3, we report a full model for the overall sample that includes all our predictors for altruistic giving (except for perceived closeness that was excluded due to its expected mediating effect) and all interaction terms. All reported effects hold in this full model demonstrating the robustness and consistency of our results.

### Additional Explorative Analyses

As we assessed participants' future anxiety (in addition to perceived competition with receiver groups) and nationalism (in addition to political left-wing orientation), we run a further exploratory analysis adding these two factors as further individual predictors to the analysis in Table 2. In the overall sample, neither future anxiety nor nationalism predicted altruistic giving (see Supplementary Table 4). However, the individual subsample analysis revealed that future anxiety was a significant negative predictor for altruistic giving toward social welfare recipients. And high level of nationalism predicted lower degrees of altruistic giving toward student refugees going beyond the effect of political orientation that remained significant in this extended analysis.

To further investigate the unexpected finding that a shared student identity increased perceived closeness but tended to decrease altruistic giving, we investigated whether increased competition in closer and more similar groups might explain this reversal. In line with the first part of this *post-hoc* explanation, participants perceived significantly more average competition toward students compared to non-students within the overall sample (*b* = 1.01, *z* = 18.96, *p* < 0.001) and when looking at the refugee (*b* = 0.36, *z* = 2.71, *p* < 0.005) and the German (*b* = 0.85, *z* = 20.62, *p* < 0.001) subsample individually (see also Supplementary Figure 2 for a graphical comparison of the individual competition measurements). Following on the prediction that perceived competition can decrease altruistic tendencies, we subsequently tested if the fact that participants perceived more competition toward students compared to non-students can account for students receiving less donations that non-students. Controlling for this increased competition toward students did, however, not account for the unexpected negative effect of shared student identity on altruistic giving (Supplementary Table 5). Hence, the negative effect on altruistic giving cannot be explained by perceived competition toward other students.

**Figure 3:**
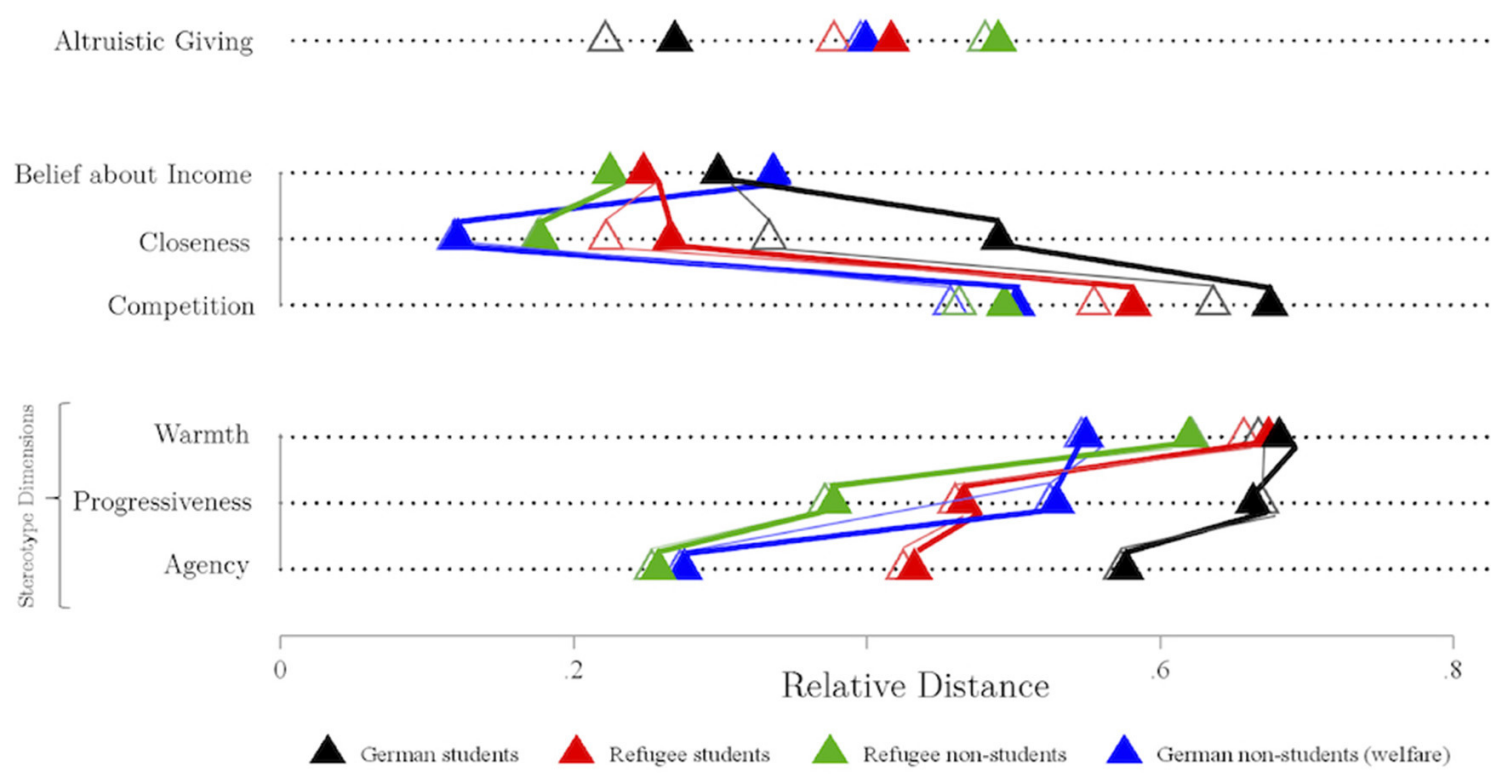
Graphical illustration of the relative distance between the individual receiver groups [German students, non-student Germans (= social welfare recipients), refugee students and non-student refugees] on predictors (belief about income, perceived closeness, competition, warmth, progressiveness, and agency) and altruistic giving. Relative distance was calculated as DG giving – min DG giving to all groups/(min DG giving to all groups – max DG giving to all groups). Full symbols mark local and hollow symbols mark non-local recipients.

To allow exploring group differences in more detail, Figure 3 presents the descriptive results for ratings on believed income, perception of closeness and competition, evaluation on the stereotype dimensions of warmth, agency and progressiveness and contact as well as the dependent variable altruistic giving separated by individual subgroups (see also Supplementary Table 6). Figure 3 illustrates interesting differences as well as (predicted) similarities between subgroups graphically plotting the relative distance between the individual receiver groups on the predictors tested. Although a visual inspection seemed to indicate that all rated dimensions relate in the groups of student and non-student refugees in a similar fashion, a statistical test showed that both groups differ on five out of eight dimensions (i.e., perceived closeness, competition, warmth, progressiveness, and agency). Interestingly, the mean ratings that differed significantly between the groups are systematically shifted toward the profile of an average German student. Nevertheless, we observed also substantial differences between German and refugee students on almost all of the measured variables except for warmth (see Supplementary Table 6). In contrast, when comparing (non-student) refugees and social welfare recipients perceived differences are much less pronounced indicating that participants perceive both groups as rather similar. Thus, it appears that social welfare recipients actually represent an adequate comparison group to investigate unique effects of refugee receivers. Both groups were rated similarly regarding perceived agency and perceived average competition. In line with this, no differences were observed on almost all of the individual competition measurements (see Supplementary Figure 2). Also contact with both groups was reported as being of similar extent. Eighty-seven percent (vs. 81%) of the participants indicated to have no contact with non-student refugees (vs. social welfare recipients).(vs. social welfare recipients). Differences are perceived regarding warmth and progressiveness as (non-student) refugees were rated as warmer but less progressive as well as having fewer financial means compared to social welfare recipients. Interestingly, even though refugees were rated as being similar or even higher on almost all of the proposed perception measures and participants assuming that they have lower financial resources, we observed no significant difference in donations toward (non-student) refugees and social welfare recipients (*b* = −17.55, *z* = −1.33, *p* = 0.183).

## Discussion

The successful integration of refugees represents a current challenge for receiving societies. In the present study, we aimed to empirically analyze the effects of potentially relevant influence factors derived from psychological theories. Specifically, we conducted a fully incentivized, pre-registered study investigating the drivers that determine host citizens' (un)willingness to engage in altruistic behavior toward refugees. As it is often the small acts of kindness that create an atmosphere of welcome and appreciation facilitating inter-cultural interactions and integration (Fussell, 2014), we investigated participants' willingness to make donations for little Christmas presents for refugees, social welfare recipients (as a German comparison group) as well as fellow students. We were particularly interested in the effect of different forms of shared identities with refugees in terms of living environment (shared local identity), social status (shared student identity) and income inequality (shared socio-economic status) on altruistic giving. We furthermore investigated the channel through which these factors function by investigating perceived closeness as potential mediator and to what extent perceived receiver characteristics and characteristics of the help giver influence the degree of altruism (toward refugees). Our study confirmed for the majority of the theoretically derived factors that they are robust predictors for altruistic giving behavior in general and have predictive value specifically for behavior toward refugees. Still, the influence of some factors could not be supported.

We showed that a shared local identity—i.e., living in the same city—influenced the decision to give as participants behaved more altruistically toward refugees that live in the same city compared to those who live in another German city. Relatedly, we found that increased perception of closeness and warmth, which typically goes along with a shared identity, predicted general and refugee giving significantly. Next to these perceived receiver characteristics, we identified givers' political and prosocial orientation to be important for altruistic giving in general and for refugee giving in particular. Replicating and extending previous results (Bajrami et al., 2017; Böhm et al., 2018), we showed that individuals holding a political left-wing (vs. right-wing) orientation were significantly more (vs. less) willing to engage in altruistic behavior toward refugees. Similarly, individuals holding a general prosocial orientation also gave more money to refugees. This result also contributes to the controversy about whether individuals' prosocial orientation is rather universal (Thielmann and Böhm, 2016; Böhm et al., 2018; Fiedler et al., 2018) or bounded by group membership leading to high prosociality toward in-group members but particularly low prosocial behavior toward out-group members (De Dreu, 2010; Abbink et al., 2012; De Dreu et al., 2015). In the context of refugee giving our data clearly support the account postulating general prosociality.

Individual receiver group analysis showed that none of the hypothesized factors (except for general prosocial orientation) predicted donations toward social welfare recipients. Consequently, we can assume that the confirmed predictors of altruism presented above are particularly relevant and—at least to some degree—unique for refugee giving as they do not predict giving to the German comparison group that share relevant characteristics with the refugees' population in terms of social and economic status.

Some of the factors that we expected to increase host citizens' willingness to display altruistic behavior did not have the hypothesized effect. With regard to a shared student identity our results actually showed a significant negative effect as students received significantly less donations than non-students. Hence, our results also show that theoretically derived predictions (in this case from social identity theory) do not hold universally in that highlighting feature of a shared group identity for a clearly relevant group (i.e., students) does not necessary lead to more positive behavior. To understand this result, it needs to be taken into account that making a shared student identity salient not only increased perceived closeness but also led to an increase in perceived competition such as an, albeit slight, increase in income estimate (i.e., decrease income differences). Surprisingly, however, both factors failed to explain this unexpected result when including them as control factors in the analysis. Consequently, there must be other factors not considered in this study driving the effect of students getting less donations than non-students. Possibly, the mere label (college) “students” is used as a cue for a privileged status that in turn signals the lack of need for support. Indeed, the public perceptions of higher education is determined by certain benefits (e.g., social benefits) college students enjoy and most importantly future success (Hu et al., 2011).

In line with the theoretical prediction of people being generally inequality averse (e.g., Fehr and Schmidt, 1999; Van Lange, 1999) we found support for the effect of perceived income differences (shared economic status) on altruistic behavior in the overall sample. This result, however, should be interpreted cautiously since the overall analysis does not allow to control for (pro)social orientation (measured as in-group giving). When rerunning the analysis excluding German students and controlling for in-group giving the effect was no longer significant. One explanation for this unclear result is our methodical proceeding. Even though lab experiments have shown that people also react to differences in income earned in economic games in the lab (e.g., Korenok et al., 2012), we cannot rule out that a small gift of 2.00€ was not sufficient to activate inequality motives since it might be negligible compared to the wealth difference in naturally occurring incomes. Future studies might use more substantial amounts of money to investigate the link between income differences and altruistic giving in the lab. For the refugee subsample, we did not find the effect of perceived income differences on altruistic behavior. Perceiving the help receiver as particularly vulnerable and in need of support might be more decisive for donation decisions (toward refugees) than estimated inequality in incomes, which is not directly taken into account in the mentioned theories. The amount of money all groups have available on a monthly basis was comparable in absolute terms as students' current financial resources do not differ significantly from government support for refugees or social welfare recipients. Nevertheless, we can assume that both refugees and social welfare recipients are perceived as socially vulnerable groups in society and thus more in need of help than (German) fellow students. This perceived neediness might activate moral norms of prosociality outweighing the effect of inequality aversion and a shared identity (regarding economic and student status) on altruistic behavior toward refugees. Indeed, previous research suggests that individuals tend to give more money when the receiving target is perceived as vulnerable and in need of help (Fisher and Ma, 2014; Malti et al., 2016; Kogut et al., 2018; Paulus, 2020). In line with these findings, Brañas-Garza (2007) showed that making the receivers' neediness salient led to increased DG allocations. By adding the sentence “Note that your recipient relies on you” to the neutral instructions, dictators became aware of the others' powerlessness, which activated a universal moral rule of helping. Our findings would also be in line with the assumption that altruistic behavior is primarily driven by moral preferences, rather than by social preferences (see Capraro and Perc, 2021, for a review). For instance, Capraro and Rand (2018) demonstrated that framing an action as morally right led to an increase in DG allocations, which could best be explained by a generalized morality preference rather than preferences for equity and/or efficiency per se. In line with the morality argument, it has been shown that moral suasion can decrease in-group favoritism in DG's (Bilancini et al., 2020). Studies on refugee helping support this line of reasoning as a large scale study including data of 15 European host countries reveals that asylum seekers who have severe vulnerabilities and are in urgent need of help received the greatest public support (Bansak et al., 2016).

The results concerning the expected mediation of perceived closeness for the effects of various manipulations of shared identity on altruistic giving are mainly not supported and the remaining effects seem to be either small or not very robust. For shared student identity and shared economic status clearly no mediation could be observed since the expected main effects of both factors (the effect of shared student identity on altruistic giving and the effect of shared economic status on perceived closeness) were in the opposite direction than expected. With regard to the effect of shared local identity on altruistic behavior, we found the expected mediating influence of perceived closeness. Thus, perceived closeness explains (at least partially) the relationship between local identity and altruistic behavior. However, this mediating effect was not robust as it did not hold when controlling for in-group giving in the overall analysis nor when restricting the analysis to refugee receivers leaving room for discussion. It can be assumed that the information of shared living environment per se triggers helping behavior toward local others. Research shows a general preference for local (compared to non-local) charity organization (Hall et al., 2013). In addition to the arguments relating to increased transparency and accountability, it was shown that individuals experience donating to local charities as more rewarding as they were more likely to see the positive impact on their local community. Local charities are typically successful through the use of strategies that increase community engagement. Thus, whether our finding of increased donations toward local (compared to non-local) refugees could be explained through increased sense of personal responsibility to help local (compared to non-local) refugees could be an interesting question for future research.

Furthermore, our results revealed no evidence for a negative effect of perceived competition on altruistic giving toward refugees, which we initially assumed based on predictions from realistic group conflict theory and previous findings explaining host society's anti-immigrant attitudes through increased perception of competition toward immigrants (e.g., Semyonov et al., 2006; Meuleman et al., 2009; Gorodzeisky, 2011). The difference might be due to the form of helping behavior we assessed in our study. Jackson and Esses (2000) showed that perceived economic competition with immigrants reduced support only for empowerment forms of help but did not affect the host society's endorsement of (non-empowering) direct assistance. In the present study we measured small acts of kindness through donations, which reached refugees in form of little presents. However, we did not measure support for empowerment forms of help that would increase refugees' competitiveness. In hindsight, we need to admit that the measure we used might not have been optimal to investigate the link between altruistic giving and perceived intergroup competition. Hence, for future studies, it would be interesting to systematically investigate the varying impact of perceived competition on empowerment vs. non-empowering forms of help toward refugees.

One caveat of the current study is that we involved student participants only. While this seems sufficient for testing theoretical predictions, results have to be used cautiously when aiming to derive policy implications for the general public. Students represent a homogenous group that typically hold rather progressive than conservative beliefs when it comes to immigration and refugees compared to the average host citizen. Hence, future research using representative sample is warranted. Given the specifics of the sample, one finding that we consider particularly interesting, is that (student) participants did not donate more money to refugees compared to welfare recipients even though the former were rated more positively on many of the receiver characteristics than the latter. Surprisingly, participants reported to feel more closeness toward (non-student) refugees than toward (German) social welfare recipients they share their cultural background (same language, cultural values etc.) with. Quite in line with this, refugees received higher ratings on the stereotype dimension of warmth, contradicting research on immigrant stereotypes that depicts the generic immigrant as rather low in warmth (and competence) (Fiske et al., 2002). However, despite this positive perception, refugees did not receive more donations. This is especially noteworthy since refugees were also evaluated to have lower financial resources compared to social welfare recipients. This finding contradicts the assumption of a general refugee bonus with the label “refugee” evoking per se higher willingness to help due to specific norms associated with refugee helping (see Böhm et al., 2018).

Our findings still provide insights for policy makers and other professionals who wish to promote positive attitudes and behaviors toward refugees to facilitate a successful integration. Highlighting refugees as part of the local communities might be an effective way to increase their willingness to help refugees. This might be accomplished by the development of local rather than national programs for refugee integration that particularly emphasize a shared sense of belonging to the same community. Such campaigns should highlight the emotional and symbolic value of the local community (e.g., a city or a neighborhood) and explicitly appeal to the individual responsibility for its members. For instance, neighborhood initiatives or local mentoring programs might provide an excellent platform for refugee integration. Naturally, this would also increase the perception of closeness toward refugees, a further factor that we identified to be predictive for altruistic behavior toward refugees. Given the correlation between perceived closeness and contact to refugees (see Supplementary Table 7), perception of closeness could be increased by promoting (positive) contact between members of the host society and refugees. This seems to be particularly important since positive (negative) contact facilitates (inhibits) positive refugee-receiving community relations (Kotzur et al., 2018; Lutterbach and Beelmann, 2020) as would be expected from intergroup contact theory (Allport, 1954; Pettigrew, 1998). In line with our results, Seethaler-Wari (2018) point out the importance of local factors in urban planning assuming that refugee integration happens on the local level of the city and the neighborhood. Consequently, policy approaches that avoid the creation of marginalized communities (e.g., refugee camps outside the city) and prioritize the dispersion of refugees (e.g., quota systems) might represent future-oriented approaches to foster integration through increased contact.

Not only contact to the refugee group but also individuals' general tendency for prosocial behavior has been identified as a key factor for altruistic giving toward refugees within this study. Hence, society at large would not just benefit from promoting prosocial and cooperative orientations of its citizens because it allows a better functioning co-existence of its citizens but also increases prosocial behavior toward new members of society. Early mentoring programs that have shown to increase general prosocial orientation in children (Kosse et al., 2020) seems also particularly effective for facilitating cross-cultural behavior among the youngest members of society.

As a caveat of the current study, it has to be considered that the generalizability and external validity of the results might be limited as behavior in the lab may not extend to behavior outside of the lab. The DG represents an artificial scenario where dictators share their “income” with receivers. In a naturally-occurring setting outside the lab very few people would, for example, give 50% of their income or wealth to refugees as they did under lab conditions in the present study. Hence, the above mentioned practical implications should be regarded as forward-looking suggestions that need further investigation in more externally valid field settings.

Overall, the present study contributes to a deeper understanding of the factors that promote host citizens' helping behavior toward refugees. The obtained results provide empirically based insights for ideas that facilitate successful refugee integration and inter-cultural co-existence. However, further research is needed to test these ideas systematically in more externally valid settings.

## Data Availability Statement

The datasets presented in this study can be found in online repositories. The names of the repository and accession number(s) can be found below: https://osf.io/mb2wj/.

## Ethics Statement

This study was reviewed and approved by the general Max Planck Block for the economic incentivized Experiments. The participants provided their written informed consent to participate in this study.

## Author's Note

Parts of the research were conducted when AG was affiliated with the University of Göttingen and when DH was affiliated with the Max Planck Institute for Research on Collective Goods, Bonn, Germany.

## Author Contributions

DH, SF, and AG contributed to conception and design of the study. DH performed the experiment and wrote the manuscript. DH and SF analyzed and interpreted the data. AG and SF supervised the preparation of the manuscript. All authors read and approved the submitted version.

## Conflict of Interest

The authors declare that the research was conducted in the absence of any commercial or financial relationships that could be construed as a potential conflict of interest.

## Publisher's Note

All claims expressed in this article are solely those of the authors and do not necessarily represent those of their affiliated organizations, or those of the publisher, the editors and the reviewers. Any product that may be evaluated in this article, or claim that may be made by its manufacturer, is not guaranteed or endorsed by the publisher.

## Footnotes

^1^Note that for the sake of comprehensibility and textual stringency we changed hypothesis ordering. We further made modifications to the pre-registered analyses adding appropriate control variables which we missed to pre-register. All changes are reported transparently. The results of the pre-registered analyses are reported in the Supplementary Materials.

^2^Since power analyses for mixed effects models cannot be calculated with G*power we used a repeated measurement ANOVA as closest approximation instead with the following specification derived from the data: Total sample size = 196, number of groups = 6, number of measurements = 4, correlation among repeated measures = 0.2, non-sphericity correction: = 0.75.

^3^Overall, 208 Participants were invited for the experiment. The pre-registered minimum sample size after exclusions (due to missing values) until which we planned to continue data collection was 189. We stopped data collection after 196 participants took part in the study and then checked if participants had to be excluded. No exclusions were necessary. Since we had no fewer than 63 participants in either condition, we did not schedule additional sessions.

^4^We chose Cologne (a city 30 km from Bonn) as “other” city and gave the participants' donations to the respective groups living in Cologne. Note that participants did not know which other German city we referred to (in order to avoid the activation of stereotypes or individual sympathy/antipathy).

^5^In the German subsample, German students were not excluded. Accordingly, this subgroup analysis does not control for general (pro)social orientation.

^6^Note that we additionally included refugee, student identity, refugee*student identity and the respective other predictor (local identity/ perceived income differences) as control variables, which we missed to pre-register but have to be included for logical reasons as well. Results do not change qualitatively when running the pre-registered analysis including only in-group giving in the analysis.
