# Supplementary material for: Altruistic Giving Toward Refugees: Identifying Factors That Increase Citizens' Willingness to Help
Source: Front Psychol. 2021 Aug 9;12:689184. doi: 10.3389/fpsyg.2021.689184 (PMC8381334; doi:10.3389/fpsyg.2021.689184)
Supplement: Supplementary file 1 [file Table_1.pdf]

Supplementary Material

Supplementary Tables

Table S1a

Altruistic giving predicted by the main predictors shared local identity and shared student identity (column 1), perceived income differences (column 2) and closeness (column 3) and all main predictors (column 4) for the overall sample including all interaction terms.

	(1)	(2)	(3)	(4)
Shared local identity	6.45*	6.45*	5.57*	5.73*
(*yes=1*)	(2.30)	(2.34)	(1.90)	(1.98)
Shared student identity	-22.20$^{[***]}$	-24.16$^{[***]}$	-28.78$^{[***]}$	-29.50$^{[***]}$
(*yes=1*)	(-5.85)	(-6.38)	(-5.81)	(-6.02)
Perceived income diff		0.04***		0.04**
		(3.24)		(3.01)
Perceived closeness			3.194*	2.73*
			(2.33)	(2.01)
Refugee	31.20***	27.52***	32.30***	28.86***
(*yes=1*)	(8.23)	(6.85)	(7.73)	(6.53)
Shared student identity*	5.56	6.11	8.50	8.13
Refugee	(0.70)	(0.75)	(0.99)	(0.93)
Shared local identity*	-0.54	-1.24	-0.51	-1.47
Refugee	(-0.09)	(-0.20)	(-0.08)	(-0.23)
Shared local identity*	7.65	7.56	7.30	7.52
Shared student identity	(1.25)	(1.25)	(1.06)	(1.11)
Shared student identity*		0.01		0.01
income diff		(0.92)		(0.88)
Shared local identity*		0.01		0.01
income diff		(0.52)		(0.56)
Refugee*		-0.02		-0.02
income diff		(-1.31)		(-1.29)
Shared student identity*			-4.20	-3.54
closeness			(-1.40)	(-1.18)
Shared local identity*			-0.52	-0.62
closeness			(-0.25)	(-0.30)
Refugee*			1.52	1.62
Perceived closeness			(0.55)	(0.59)
Constant	67.51***	68.11***	69.51***	69.86***
	(16.88)	(16.75)	(16.92)	(16.79)
Observations	784	784	784	784

Numbers refer to unstandardized regression coefficients for the predictor variables (all predictor variables were centered for the analysis and before calculating the interaction terms), *z* statistics in parentheses, * $p < .05$, ** $p < .005$, *** $p < .001$, all stars indicate one-sided test results, if effects are in the opposite direction to the predicted hypotheses stars are reported in brackets “[]” for informative reasons only.

Table S1b

Altruistic giving predicted by the main predictors shared local identity and shared student identity (column 1), perceived income differences (column 2) and closeness (column 3) and all main predictors (column 4) for the overall sample excluding German students.

	(1)	(2)	(3)	(4)
Predictors:				
Shared local	3.43*	3.43*	3.08	3.08
Identity (*yes=1*)	(1.78)	(1.78)	(1.61)	(1.61)
Shared student	-16.72	-16.74	-18.21[*]	-18.31[*]
Identity (*yes=1*)	(-1.54)	(-1.54)	(-1.68)	(-1.68)
Perceived		-0.01		-0.01
income diff		(-0.02)		(-0.12)
Perceived			3.13*	3.14*
closeness			(2.14)	(2.14)
Controls:				
Refugee	23.72*	23.78*	22.44*	22.82*
(*yes=1*)	(2.18)	(2.10)	(2.07)	(2.02)
Prosocial	0.65***	0.65***	0.64***	0.64***
orientation	(8.42)	(8.42)	(8.41)	(8.41)
(in-group giving)				
Constant	38.62***	38.61***	33.27***	33.14***
	(4.27)	(4.24)	(3.56)	(3.52)
Observations	392	392	392	392

Numbers refer to unstandardized regression coefficients for the predictor variables, *z* statistics in parentheses, * $p < .05$, *** $p < .001$, all stars indicate one-sided test results, if effects are in the opposite direction to the predicted hypotheses stars are reported in brackets “[]” for informative reasons only.

Table S2

Perceived closeness predicted by shared local identity, shared student identity, and perceived income differences for the overall (all receivers, column 1), the refugee (student and non-student refugees, column 2) and the German (German students and social welfare recipients) sample and for each individual receiver group separately (column 4-7).

	Overall	Refugees	Germans	German students	Student Refugees	Non-student Refugee	Welfare recipients
Predictors:							
Shared local identity (*yes=1*)	0.60*** (6.96)	0.16 (1.61)	0.83*** (7.77)	1.10*** (9.68)	0.31* (2.18)	0.02 (0.11)	0.02 (0.09)
Shared student identity (*yes=1*)	1.41*** (12.89)	0.50* (2.54)	1.97*** (14.17)				
Perceived income diff	0.01** (2.97)	0.01 (1.03)	0.01+ (1.90)	0.01 (1.62)	0.01 (1.51)	0.01 (0.04)	0.01* (2.03)
Controls:							
Refugee (*yes=1*)	-0.73*** (-6.47)						
Prosocial orientation (in-group giving)		0.01 (1.42)			0.01 (1.46)	0.01 (0.64)	-0.01 (-0.78)
Constant	2.05*** (16.33)	1.98*** (11.05)	1.49*** (10.08)	3.33*** (30.13)	2.27*** (10.00)	2.16*** (11.88)	1.96*** (10.65)
Observations	784	262	522	392	130	132	130

Numbers refer to unstandardized regression coefficients for the predictor variables, *z* statistics in parentheses, $^{+}$ $p < .10$, * $p < .05$, *** $p < .001$, all stars indicate two-sided test results.

Table S3

Full model: Altruistic giving predicted by all predictors including all interactions for the dummy variables (column 1) and the continuous variables (column 2)

	(1)		(2)	
Perceived competition	1.36	(0.37)	2.20	(0.61)
Perceived warmth	5.95***	(3.81)	5.02**	(3.20)
Political orientation *(left=0, right=10)*	-7.67**	(-3.17)	-7.39**	(-3.07)
Shared local identity (*yes=1*)	5.64*	(2.02)	5.23*	(1.90)
Shared student identity (*yes=1*)	-30.78[***]	(-6.49)	-30.36[***]	(-5.83)
Refugee (*yes=1*)	25.51***	(6.43)	23.39***	(5.53)
Perceived income diff	0.04***	(3.67)	0.04***	(3.52)
Refugee*shared local identity	-0.46	(-0.08)	-2.04	(-0.33)
Refugee*shared student identity	13.04	(1.62)	7.50	(0.82)
Shared local identity*student identity	6.84	(1.14)	7.92	(1.15)
Refugee*competition			2.80	(0.57)
Refugee*warmth			9.47***	(3.57)
Refugee*political orientation			-7.59**	(-3.09)
Refugee*income diff			0.01	(0.29)
Refugee*closeness			-1.84	(-0.70)
Shared local identity*competition			-1.31	(-0.36)
Shared local identity*warmth			0.97	(0.50)
Shared local identity*political orientation			1.94	(1.16)
Shared local identity*income_diff			0.01	(0.66)
Shared local identity*closeness			-0.96	(-0.46)
Shared student identity*competition			-0.32	(-0.06)
Shared student identity*warmth			-3.57	(-1.22)
Shared student identity*political orientation			3.47	(1.45)
Shared student identity*income diff			0.01	(0.09)
Shared student identity*closeness			1.71	(0.58)
Constant	67.93***	(17.38)	67.48***	(16.43)
Observations	784	784	784	784

Numbers refer to unstandardized regression coefficients for the predictor variables (all predictor variables were centered for the analysis and before calculating the interaction terms), *z* statistics in parentheses, * $p < .05$, ** $p < .005$, *** $p < .001$, all stars indicate one-sided test results, if effects are in the opposite direction to the predicted hypotheses stars are reported in brackets “[]” for informative reasons only.

Table S4

Altruistic giving predicted by future anxiety, nationalism, perceived competition, perceived warmth, political left-wing orientation and prosocial orientation for the overall (all receivers, column 1), refugee (student and non-student refugees, column 2) and German (German students and social welfare recipients) sample and for each individual receiver group separately (column 4-7).

	Overall	Refugees	Germans	German students	Student Refugees	Non-student Refugee	Welfare recipients
Predictors:							
Future anxiety	-2.29 (-0.38)	4.80 (0.64)	-5.15 (-0.88)	-1.33 (-0.23)	1.95 (0.20)	10.04 (0.89)	-36.52** (-3.16)
Nationalism	-4.41 (-1.05)	-7.93 (-1.51)	-1.73 (-0.43)	-4.01 (-0.98)	-14.55* (-2.33)	1.69 (0.19)	13.05 (1.62)
Perceived competition	0.46 (0.13)	4.74 (0.88)	3.49 (0.87)	4.34 (1.04)	3.44 (0.43)	-1.28 (-0.27)	6.42 (0.88)
Perceived warmth	5.77*** (3.72)	10.90*** (3.94)	0.61 (0.34)	1.80 (0.88)	8.72* (1.95)	6.72** (2.71)	1.82 (0.39)
Political Orientation[a]	-6.99*** (-2.82)	-10.74*** (-3.40)	-4.19* (-1.74)	-3.24 (-1.33)	-8.43* (-1.84)	-13.50** (-2.94)	-4.56 (-0.99)
Prosocial orientation		0.67*** (7.96)			0.64*** (5.92)	0.65*** (4.95)	0.58*** (4.75)
Controls:							
Shared local identity (*yes=1*)	5.79* (2.07)	3.41 (1.46)	6.65* (2.28)	8.53*** (4.01)	5.97 (1.37)	2.06 (1.32)	-0.51 (-0.13)
Shared student identity (*yes=1*)	-29.82[***] (-6.30)	-24.03[*] (-2.32)	-30.08[***] (-5.38)				
Refugee (*yes=1*)	25.48*** (6.41)						
Constant	78.65** (3.26)	21.54 (0.61)	94.73*** (3.95)	43.58+ (1.67)	36.70 (0.78)	41.18 (0.88)	97.51+ (1.78)
Observations	784	262	522	392	130	132	130

Numbers refer to unstandardized regression coefficients for the predictor variables, *z* statistics in parentheses, $^{+}$ $p < .10$, * $p < .05$, ** $p < .005$, *** $p < .001$, all stars indicate one-sided test results, if effects are in the opposite direction to the predicted hypotheses stars are reported in brackets "[]" for informative reasons only; [a] higher values indicate more right-wing orientation.

Table S5

Altruistic giving predicted by perceived competition, shared local identity, shared student identity for the overall (all receivers, column 1), refugee (student and non-student refugees, column 2) and German (German students and social welfare recipients) sample.

	Overall	Refugees	Germans
Predictors:			
Perceived competition	0.01<br>(0.00)	0.28<br>(0.05)	2.11<br>(0.54)
Shared local identity (*yes=1*)	6.45*<br>(2.26)	4.75*<br>(2.05)	6.94*<br>(2.36)
Shared student identity (*yes=1*)	-22.21$^{[***]}$<br>(-4.86)	-16.76<br>(1.49)	-25.54$^{[***]}$<br>(-4.97)
Controls:			
Refugee (*yes=1*)	31.20***<br>(8.15)		
Refugee* Shared student identity	5.57<br>(0.68)		
Prosocial orientation (in-group giving)		0.69***<br>(7.19)	
Constant	67.51***<br>(16.87)	88.26***<br>(15.98)	55.03***<br>(14.48)
Observations	784	262	522

Numbers refer to unstandardized regression coefficients for the predictor variables (all predictor variables were centered for the analysis and before calculating the interaction term refugee*shared student identity), z statistics in parentheses, * $p < .05$, *** $p < .001$, all stars indicate one-sided test results, if effects are in the opposite direction to the predicted hypotheses stars are reported in brackets “[]” for informative reasons only.

Table S6

Ratings on altruistic giving, belief about income, perception on closeness, competition, warmth, progressiveness, agency and contact separated by individual subgroups.

					Subsample comparisons			
	German students	Student refugees	Nonstudent refugees	Welfare recipients	German students vs. Student refugees	Nonstudent refugees vs. Welfare recipients	Nonstudent refugees vs. Student refugees	German students vs. Welfare recipients
Altruistic giving	24.52%	39.69%	48.50%	39.73%	16.13*** (5.28)	8.77 (1.33)	-8.811 (-1.35)	-13.33*** (-4.02)
Belief about income[a]	447.88 165.68	371.23 148.34	337.18 168.53	504.02 225.53	-88.95*** (-5.57)	-166.80*** (-4.84)	34.05 (1.24)	-58.50* (-2.38)
Perceived closeness	3.88 1.34	2.71 1.27	2.23 0.98	1.84 0.93	-1.22*** (-8.36)	0.40* (2.39)	0.473* (2.40)	2.02*** (12.37)
Perceived competition	3.62 0.72	3.27 0.76	2.91 0.77	2.92 0.63	-0.26*** (-6.68)	-0.01 (-0.04)	0.359** (2.71)	0.82*** (12.68)
Perceived warmth	7.74 1.33	7.65 1.31	7.20 1.69	6.48 1.31	-0.10 (-0.93)	0.73** (2.77)	0.449^{+} (1.71)	1.21*** (7.15)
Perceived progressiveness	7.66 1.35	5.63 1.79	4.74 1.68	6.27 1.33	-2.03*** (-9.67)	-1.53*** (-5.82)	0.888** (2.95)	1.39*** (7.25)
Perceived agency	6.75 1.40	5.28 1.66	3.55 1.15	3.74 1.37	-1.56*** (-9.86)	-0.19 (-0.85)	1.732*** (7.01)	2.94*** (16.07)
Contact[b]	94%	8%	13%	19%	-0.87*** (-34.26)	-0.06 (-1.47)	-0.0519 (-1.41)	0.75*** (25.58)
Observations	196	65	66	65	261	131	131	261

t statistics in parentheses, $^{+}$ $p < .10$, * $p < .05$, ** $p < .005$, *** $p < .001$, all stars indicate two-sided test results,

[a] Participants indicated their beliefs about the monthly net income of members from different social groups on the basis of 21 categories ranging from 250 € to 5000€ in steps of 250€ (<250€, 250-500€… >5001€). For each category, we used the respective mean (e.g., for the category 250-500€, M = 375€).

[b] We measured contact by asking participants “Do you have frequent contact with...?” (Yes/No).

Table S7

Correlation of all predictors

	Shared local identity[a]	Shared student identity[a]	Perceived income diff[a]	Perceived closeness[a]	Perceived competition[a]	Perceived warmth[a]	Perceived agency[a]	Perceived progressiveness[a]	Contact[a]	Political orientation[b]	Social orientation	Nationa-lism
Shared student identity	0.01											
Perceived income diff	0.01	-0.03										
Perceived closeness	0.18***	0.43***	0.06									
Perceived competition	0.09	0.36***	0.02	0.23***								
Perceived warmth	0.03	0.28***	-0.01	0.32***	0.07							
Perceived agency	0.01	0.65***	-0.03	0.40***	0.32***	0.24***						
Perceived progressiveness	0.01	0.41***	-0.08	0.31***	0.25***	0.16***	0.42***					
Contact	0.01	0.53***	-0.06	0.50***	0.28***	0.20***	0.53***	0.44***				
Political orientation	0.01	-0.06	0.13*	-0.05	0.15**	-0.05	0.11	0.05	-0.03			
Social orientation	0.01	-0.02	0.05	0.03	-0.05	0.02	0.03	0.03	-0.02	-0.05		
Nationalism	0.01	0.01	0.01	-0.05	0.05	0.01	0.09	0.04	-0.04	0.22**	-0.07	
Future anxiety	0.01	-0.06	-0.02	-0.10	0.23***	0.07	-0.09	-0.01	-0.01	-0.02	-0.01	0.08

[a] based on repeated measures, * $p < .05$, ** $p < .01$, *** $p < .001$. [b] higher values indicate more right-wing orientation.

Supplementary Figures

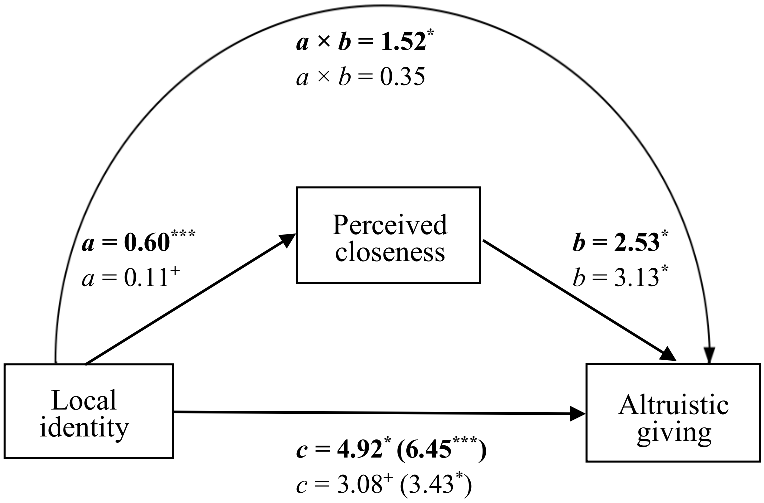

Figure S1a. Overall sample: The figure shows the effect of local identity on perceived closeness (a), the effect of perceived closeness on DG giving (b), the effect of local identity on DG giving (see the effect after controlling for perceived closeness in parentheses), and the indirect effect of local identity on DG giving via perceived closeness (a × b). For the indirect effect, standard errors are bootstrapped with 500 repetitions. Reported path values in bold are centered regression coefficients for the overall analysis that controls for shared student identity, refugee, student identity*refugee and perceived income differences. Reported path values below are centered regression coefficients for the overall sample excluding German students and additionally controlling for in-group giving $^{+}p < .10$, $^{*}p < .005$, $^{***}p < .001$, all stars indicate one-sided test results.

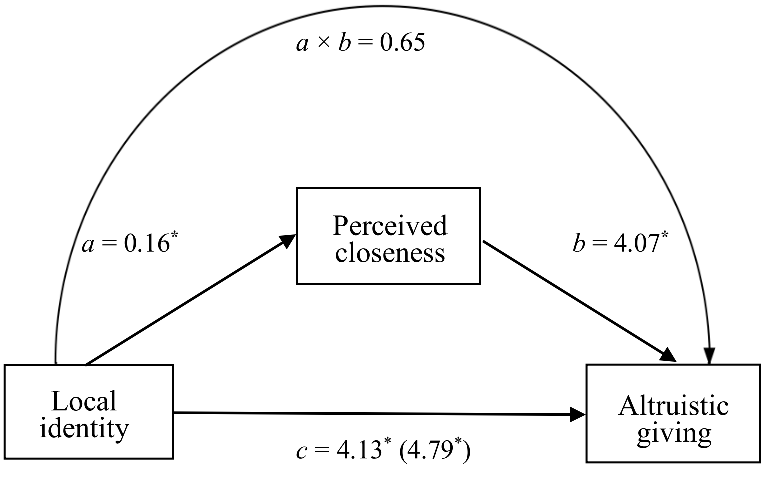

Figure S1b. Refugee subsample: The figure shows the effect of local identity on perceived closeness (a), the effect of perceived closeness on DG giving (b), the effect of local identity on DG giving (see the effect after controlling for perceived closeness in parentheses), and the indirect effect of local identity on DG giving via perceived closeness (a × b). For the indirect effect, standard errors are bootstrapped with 500 repetitions. Reported path values are unstandardized regression coefficients. $^{*}p < .05$, all stars indicate one-sided test results. The analysis controls for shared student identity, perceived income differences and in-group giving.

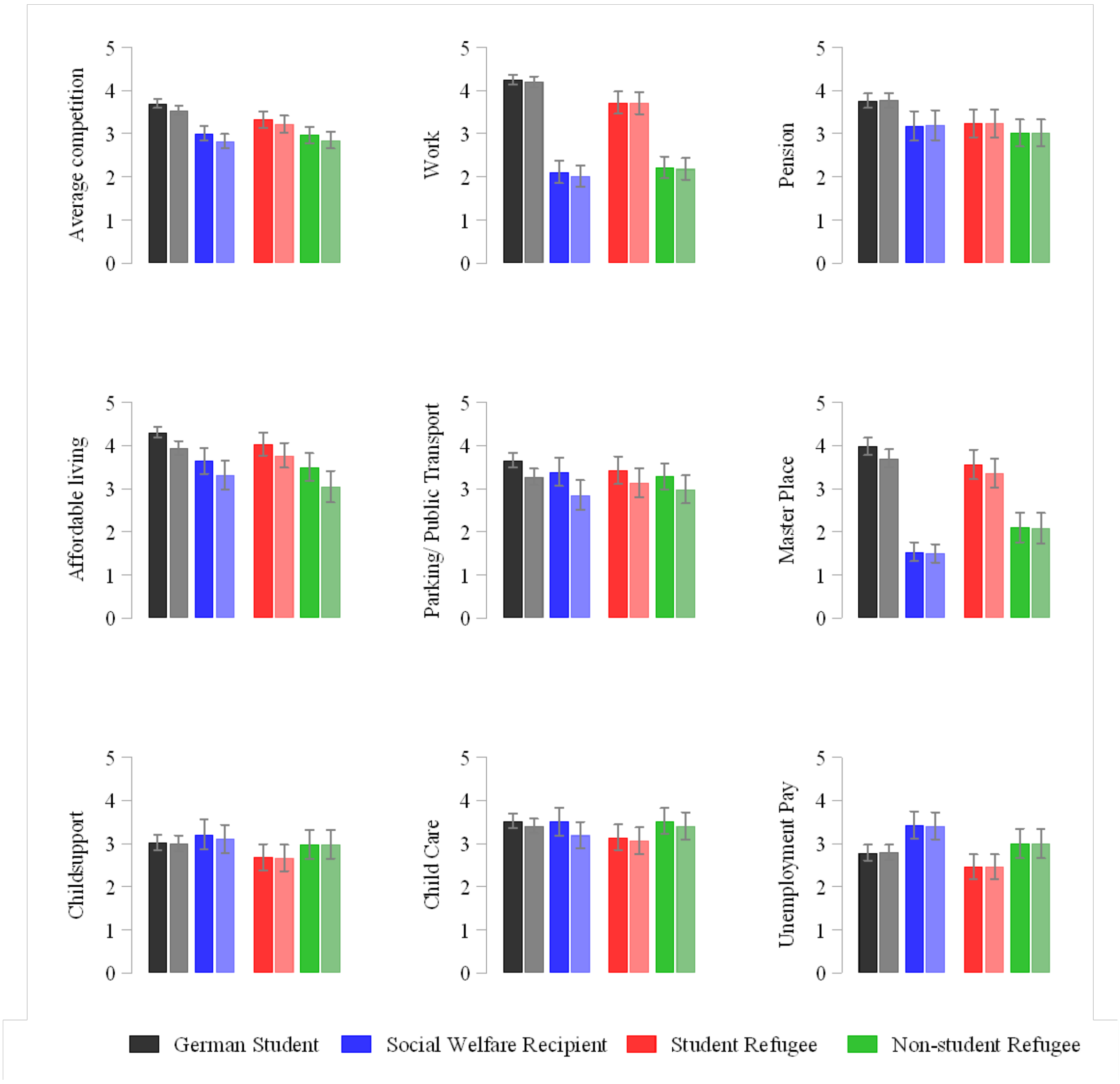

Figure S2. Histograms showing average perceived competition such as perceived competition on the eight individual competition measurements towards all subsamples of receivers. Bars in saturated colors represent the receivers with a shared local identity whereas bars in brighter colors represent nonlocal receiver groups. Error bars represent 95% confidence intervals.

Analysis as pre-registered

H1 (pre-registered H2): The effect of shared local identity:
In line with our modified analysis reported in the article and in support of the hypothesis, the pre-registered analysis (including perceived income differences and in-group giving as control variables) revealed a significant effect of shared local identity on altruistic giving (b = 6.45, z = 2.04, p < .05).

H2 (pre-registered H3): The effect of shared student identity:
In line with our modified analysis reported in the article but contrary to the hypothesis, the pre-registered analysis (including shared local identity, perceived income differences and in-group giving as control variables) revealed a significant effect of shared student identity on altruistic giving (b = -33.76, z = -10.03, p < .001) in the opposite direction as expected.

H3 (pre-registered H4): The effect of perceived income differences:
In line with our modified analysis reported in the article and in support of the hypothesis, the pre-registered analysis (including shared local identity and in-group giving as control variables) revealed a significant effect of shared local identity on altruistic giving (b = 0.03, z = 3.50, p < .001).

H4 (pre-registered H5): The effect of perceived closeness:
Contrary to our modified analysis reported in the article and contrary to the hypothesis, the pre-registered analysis (including shared local identity and in-group giving as control variables) revealed a significant effect of perceived closeness on altruistic giving (b = -4.90, z = -4.57, p < .001) in the opposite direction as expected.
Note that this is due to missing control variables (e.g., shared student identity).

H4a-c (pre-registered H6): The mediation effect of perceived closeness on...
H4a ... the effect of shared local identity on altruistic behavior
In line with our modified analysis reported in the article and in support of the hypothesis, the pre-registered analysis (including in-group giving as control variables) revealed a significant indirect effect for a shared local identity (bias-corrected CI.95[-4.54, -1.40]).

H4b ... the effect of shared student identity on altruistic behavior
Since we did not find the expected positive main effect for a shared student identity on altruistic giving the requirements for mediation analyses (e.g., Baron & Kenny, 1986) were not met (rejecting H4b).

H4c ... the effect of perceived income differences on altruistic behavior
Since we did not find the expected positive main effect for perceived income differences on perceived closeness the requirements for mediation analyses (e.g., Baron & Kenny, 1986) were not met (rejecting H4c).

H5 (pre-registered H7): The effect of perceived competition:
Contrary to our modified analysis reported in the article and contrary to the hypothesis, the pre-registered analysis (including perceived income differences and in-group giving as control variables) revealed a significant effect of perceived competition on altruistic giving (b = -14.33, z = -5.61, p < .001) in the opposite direction as expected.
Note that this is due to missing control variables (e.g., shared student identity).

H6 (pre-registered H9): The effect of perceived warmth:
In line with our modified analysis reported in the article and in support of the hypothesis, the pre-registered analysis (including shared local identity, shared student identity, refugee, perceived income differences and in-group giving as control variables) revealed a significant effect of perceived warmth on altruistic giving ($b = 5.45$, $z = 4.13$, $p < .001$).

H7 (pre-registered H1): The effect of prosocial orientation (in-group giving):
In line with our modified analysis reported in the article and in support of the hypothesis, the pre-registered analysis (including all participants and no control variables) revealed a significant effect of prosocial orientation on altruistic giving ($b = 0.76$, $z = 18.63$, $p < .001$).

H8 (pre-registered H8): The effect of political orientation (left-wing orientation)
In line with our modified analysis reported in the article and in support of the hypothesis, the pre-registered analysis (including shared local identity, shared student identity, refugee, perceived income differences and in-group giving as control variables) revealed a significant effect of political orientation on altruistic giving ($b = -5.87$, $z = -4.09$, $p < .001$).
